# Implementing the EU HTA regulation: Insights from semi-structured interviews on patient expectations, Belgian and European institutional perspectives, and industry outlooks

**DOI:** 10.3389/fphar.2024.1369508

**Published:** 2024-04-10

**Authors:** Thomas Desmet, Maud Brijs, Frank Vanderdonck, Sven Tops, Steven Simoens, Isabelle Huys

**Affiliations:** ^1^ Department of Pharmaceutical and Pharmacological Sciences, KU Leuven, Leuven, Belgium; ^2^ Healthcare Management Centre, Vlerick Business School, Ghent, Belgium; ^3^ AxTalis B.V, Gentbrugge, Belgium

**Keywords:** health technology assessment, EU HTA regulation, perspectives, semi-structured interview, health policy, joint scientific consultation, joint clinical assessment

## Abstract

**Introduction:** The goal of the Health Technology Assessment (HTA) Regulation 2021/2282 is to establish a more harmonized HTA framework, fostering member states cooperation and enabling equal patient access to innovative health technologies in Europe. This research aimed to assess the impact of the regulation on national HTAs, the strategic implications for health technology developers, and its influence on price and reimbursement negotiations.

**Methods:** A scoping literature review encompassing peer-reviewed literature as well as grey literature was conducted. Between February and March 2023, semi-structured interviews (n = 20) were performed with stakeholders from Belgian governmental institutions, European institutions, advanced therapy medicinal product developers, academics, and sickness funds. The interviews were analyzed using the framework analysis method.

**Results:** Numerous steps, such as the development of implementing acts and procedural guidelines remain to be taken. At member state level, national/regional HTA bodies and payers must act to adopt the new concepts of Joint Scientific Consultations (JSC) and Joint Clinical Assessments (JCA) within their national legislation, as well as revise their timelines and prepare for interactions at a European level. Compiling a harmonized PICO (Population, Intervention, Comparator, and Outcome), adapting local procedures, and increasing capacity to actively take part in the JSC and JCA are seen as primary barriers by several stakeholders. Training and education will help HTA bodies, payers, and health technology developers to participate in the European processes.

**Conclusion:** While practical and legal challenges were identified, recommendations (such as actively preparing for the upcoming changes and increasing capacity while providing training) were provided to adapt national and European procedures to the needs of the HTA Regulation 2021/2282. The importance of fostering collaborations and aligning local HTA procedures with the new way of working set out by the Regulation was demonstrated with this study.

## 1 Introduction

To define the added value of health technologies and make healthcare decisions, health technology assessments (HTA) are carried out. Across Europe, a heterogeneity of criteria is used to perform HTAs, resulting sometimes in inequality of patient access and variety of health technologies i.e., advanced therapy medicinal products (ATMPs), available. The heterogeneity of criteria or quality of an HTA is not always the limiting factor for patient access, yet the budgetary constraints as speakers from Eastern European countries stressed during the EPA World Congress 2023. This urges the need for a new or more harmonized HTA framework, which is exactly what the European Commission had in mind with the HTA Regulation 2021/2282 (HTAR). The HTAR aims to foster the availability of innovative health technologies to patients in Europe and to strive towards better resource management, i.e., by avoiding duplicate work at the member state (MS) level (The European Parliament and the Council of the European Union, 2021). Furthermore, it pursues to support cooperation between MS, while implementing more predictable methodologies and procedures (The European Parliament and the Council of the European Union, 2021). This should avoid discrepancies in access for patients across Europe (The European Parliament and the Council of the European Union, 2021; Natz, 2023). Therefore, the Regulation provides a Member State Coordination Group accommodating representatives from national and regional HTA bodies of each MS. This Coordination Group is responsible for coordinating the work of the subgroups and supporting the European Commission in writing the implementing acts. Whereas the European Commission will serve as a Secretariat (art. 28 of HTAR), four subgroups are defined and entitled to i) identify emerging health technologies (horizon scanning), ii) provide methodological and procedural guidelines (e.g., guidance on electing the assessors and co-assessors), iii) coordinate the Joint Scientific Consultation (JSC), and iv) oversee the Joint Clinical Assessment (JCA) (The European Parliament and the Council of the European Union, 2021).

The subgroup that will coordinate the JSC will promote the exchange of information between health technology developers (HTDs) and HTA bodies. Only health technologies of which the clinical investigations and studies are still in the planning stage are eligible for a JSC. To achieve this, the opinion of the HTD, patients, clinicians and other relevant experts will be taken into consideration during these consultations (The European Parliament and the Council of the European Union, 2021). These JSCs will cover aspects concerning the patient population, the intervention, the comparators, and the perceived health outcomes (PICO) to be later used in the JCA.

The other subgroup that will oversee the JCA focuses on the relative effectiveness assessment of health technologies. Only the clinical assessment will be conducted at the European level without passing any judgement on the overall clinical value. The purpose is that it will be a descriptive representation of i) the relative effects based on the parameters chosen in the PICO, ii) the degree of certainty of those effects, and iii) the strengths and limitations of the evidence (cfr. Article 9 of the Regulation) (The European Parliament and the Council of the European Union, 2021). Cost-effectiveness, value assessments, and pricing and reimbursement decisions will remain the authority of the individual MS. The JCA report will cover four out of nine domains as presented by the European Network for Health Technology Assessment (EUnetHTA) Core Model^®^ (EUnetHTA, 2016; EUnetHTA, 2022).

From 12 January 2025 onwards, all novel anticancer drugs and ATMPs, filing for marketing authorization via the centralized procedure of the European Medicines Agency (EMA), will be subject to a JCA. From 13 January 2028 on, it shall apply for orphan drugs, and from 13 January 2030 on all other medicinal health technologies as well as new indications of HTAR-assessed medicines (The European Parliament and the Council of the European Union, 2021). In some cases, a JCA can be performed earlier than planned i) for medicinal products that have the potential to address an unmet medical need, ii) in case of a public health emergency or iii) if it has a significant impact on the healthcare system (The European Parliament and the Council of the European Union, 2021).

As of these dates, MS are obliged to give due consideration to the published JCA report in their national decision making while assessing health technologies. Furthermore, to avoid duplication of work, MS cannot request information that was already requested at a European level. Similarly, information requested by another MS cannot be requested again as it needs to be shared in an IT platform that will be created by the European Commission.

The Beneluxa initiative initially fostered a collaboration between Belgium and the Netherlands since 2015. Later, the Grand Duchy of Luxembourg, Austria and Ireland joined. This initiative was welcomed as one of the first attempts to conduct a joint assessment, it being understood that every country still follows its local reimbursement procedures (BeNeLuxA, 2018). Next to Beneluxa, FINOSE is a HTA collaborating network encompassing a joint assessment between Finland, Norway, Sweden and recently Denmark (Norwegian Medicines Agency, 2023).

The aim of this study was to identify potential barriers and elucidate how HTA bodies, Belgian, and European institutions could prepare for the implementation of the European HTAR. Furthermore, the strategic impact for HTDs and consequences on patient access were evaluated considering the new concepts of JSC and JCA.

## 2 Methods

A scoping literature review was performed from September 1st until 23 December 2022, followed by semi-structured interviews. Additional literature was sourced through snowballing during data analysis and write-up of the manuscript.

### 2.1 Scoping literature review

A scoping literature review was performed to gain insights and a broad perspective on the topic. The literature review covered legislative, peer-reviewed, and grey literature. PubMed was used to find peer-reviewed literature, adopting the search terms “ATMP”, “HTA”, “Joint clinical assessment”, “Joint scientific”, “Europe”, “EUnetHTA”, and “PICO”. Additional terms such as “challenges”, “reimbursement”, and “Belgium” were used in the grey literature search. This first literature search was the basis for shaping the topics and questions of the interview guide. A total of 477 hits in PubMed resulted in 414 unique results. Only Dutch or English articles were included based on screening of both title and abstract, leading to 291 articles. Only articles from 2013–2022 were included, as the relevance of older articles on the current legislation was questioned. Screening on title and abstract excluded another 220 articles leaving 71 articles for inclusion. The snowballing technique has yielded an additional twelve relevant articles that complement our initial findings. For the grey literature, webpages of regulatory institutions such as EMA and national HTA agencies as well as EUnetHTA were screened.

Through a collaborative process between the authors, the interview guide was crafted to ensure alignment with the research objectives, thereby facilitating the elicitation of meaningful insights and nuanced perspectives.

### 2.2 Semi-structured interviews

Semi-structured interviews were performed with representatives of five stakeholder groups: i) Belgian governmental institutions, ii) European institutions, iii) ATMP developers, iv) academics, and v) sickness funds. Approval from the ethics committee Social-Societal Ethics Committee of the KU Leuven was obtained in February 2023 (G-2022–6137-R2). Such a type of interview allowed individual participants to express their opinions without influence from others. A semi-structured interview guide (Supplementary Material S1) was developed based on the scoping literature review and discussions with several stakeholders. A list of standardized open questions was asked to all stakeholder groups, whereas more refined questions were asked to the five individual stakeholder groups. The questions were categorized into three topics: HTAs, ATMPs and JCAs. Stakeholders were invited to participate based on their relevant background and/or involvement with HTA, the HTAR or working in the domain of market access. A broad perspective of participants with different backgrounds were included. Participants could also be contacted based on referrals by others who have already been interviewed (snowballing). Participants were excluded from the interviews if they were unfamiliar with the European Regulation, had insufficient experience or knowledge concerning market access or HTA or had insufficient knowledge of English or Dutch.

Selected participants were invited by MB via publicly available contact information, or their contact details obtained through the network of the authors. When participants expressed interest, an e-mail was sent to the participant with the informed consent form. This e-mail clearly stated that the participant can ask any question regarding the study they still have. All materials (invitation, informed consent form, and if requested the interview guide) were provided to the participant in their preferred language (Dutch or English).

The interviews took place between February 2023 and March 2023 and were conducted in person or virtually (Microsoft Teams or Zoom) depending on the preferences of the interviewee. Every interview was performed by MB with the supervision of TDS. Each interview lasted approximately 1 h, and questions were tailored to the individual experiences of each participant from the five stakeholder groups.

### 2.3 Data analysis of interviews

The audio recordings of the interviews were transcribed ad verbatim and removed after transcription. The transcripts were pseudonymized, all names and references that could lead to the identification of participants were removed and every participant received a unique identifier. Consequently, the transcripts were analyzed using framework analysis (Gale et al., 2013), a qualitative content analysis method, using Nvivo software (version 1.7.1). Complementary deductive codes, drafted based on the interview guide, and inductive codes (themes), arising throughout the process of analyzing the transcripts, were used to index the raw data. Subsequently, codes were grouped into categories of correlated topics and concepts. The resulting coding tree (Supplementary Material S2) was co-created by MB and TDS.

## 3 Results

The participants comprised two academics, four individuals associated to Belgian governmental institutions, six representatives from European institutions, six professionals from the pharmaceutical sector, and two employees working at a sickness fund. The opinions expressed by the interviewees are personal views and may not necessarily represent the view of the organization they are related to.

### 3.1 Expected/preliminary timelines for HTAR implementation

**FIGURE 1:**
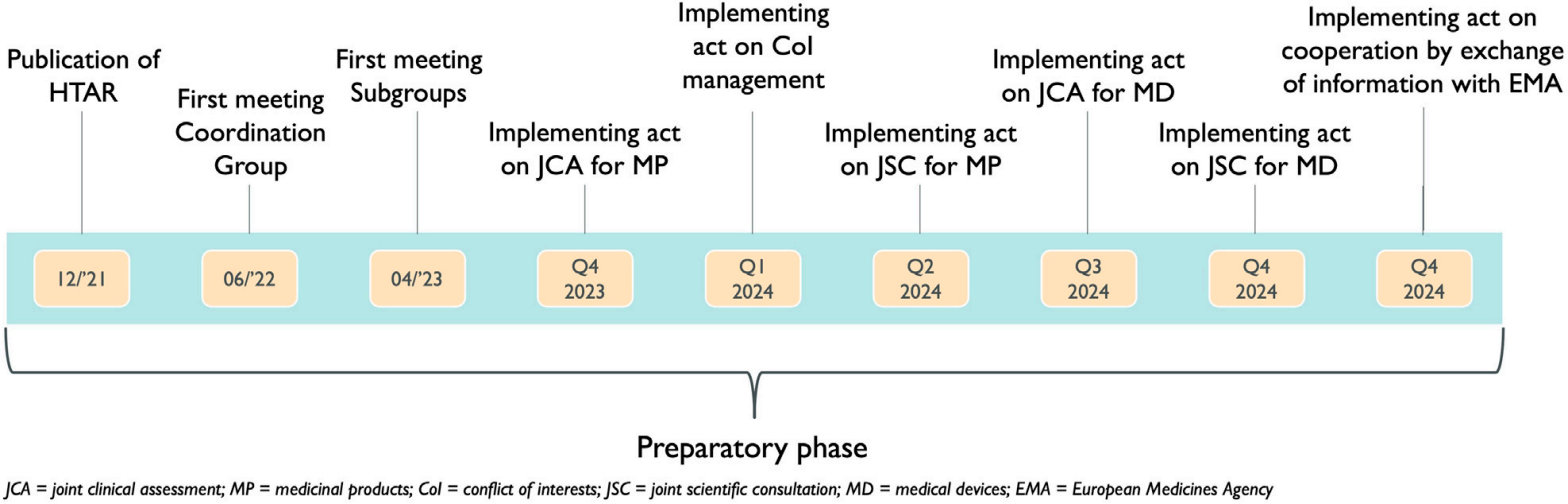
Timing of the implementing acts already published or to be published during the preparatory phase of the Health Technology Assessment Regulation.

The HTAR was published in December 2021. From this moment on, several phases of the preparatory phase have been started. In July 2022 the first Coordination Group meeting took place, followed by the inaugural meeting of the Subgroups in April 2023 (European Union, 2023). Six implementing acts remain to be published by the European Commission and by December 2024 all implementing acts ought to be published (Figure 1) (Directorate-General for Health and Food Safety, 2023a).

During the interviews, it became clear that all participants were eagerly waiting to discover the content of the implementing acts because these will describe the procedures of both the JSC and the JCA. Starting from 5 March 2024, the European Commission initiated an online public consultation regarding the draft Implementing Act on JCAs of Medicinal Products. This consultation will continue accepting responses until 2 April 2024 (European Commissiona; European Commissionb). However, one stakeholder stressed that all MS should already internally discuss the implementation of the HTAR at the national or local level in parallel with the activities performed by the subgroups. Examples of issues raised during the interviews that still need to be discussed include the designation of assessors, training programs (for assessors), more agreement on the methodologies for HTA used, and consolidation of the joint PICO.

### 3.2 Joint scientific consultation

“*The coordination group shall carry out joint scientific consultations in order to exchange information with HTDs on their development plans for a given health technology*” (The European Parliament and the Council of the European Union, 2021).

Generally, the concept of JSC was welcomed positively by most stakeholders. However, several critical points were brought up during interviews such as the number of consolidated PICOs to be considered as well as possibly limited resources which can result in a limited number of JSCs. On top of that, there was consensus that a JSC should be held as early as possible in the drug life cycle.

Interviewees highlighted that the exact procedure to select eligible health technologies for a JSC as well as the timeframe for these JSCs remain to be decided. The precise details of the JSC remain to be put into an implementing act which is expected by Q2 of 2024 (Figure 1).

Regarding the necessary type of data to perform a European HTA, different opinions were found among the interviewees. While some underlined the importance of relying on existing data, others stressed data collection should also be adapted to the specific data requirements of payers. Regarding the latter, some interviewees cautioned against requesting data that might be challenging to collect, generate, or provide.

**FIGURE 2:**
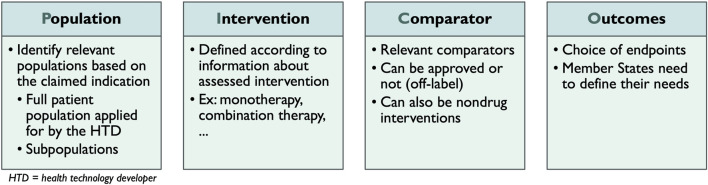
Interpretation of the concept of PICO.

All stakeholders were neutral to positive regarding the concept of a PICO (Figure 2). Nevertheless, since all MS are supposed to provide their PICO, several stakeholders stressed the importance of limiting the number of final PICOs. Stakeholders explained that the standard of care for a particular disease is not the same across MS. Consequently, this concern relates mainly to the concept of the comparator C) in the PICO. “*We must be careful that it does not become a many-headed monster.”*

### 3.3 Joint Clinical Assessment

“*I think that’s definitely a step forward to support innovation and consistency and similar access to all patients in Europe*.”

Across the different interviewees, the sentiment about the JCA was positive, mindful that its success will depend on the actual implementation and its procedures.

The JCA will only cover the clinical assessment of health technologies. To be able to give due consideration, MS should include the JCA procedures in their national HTA framework, along with their pricing and reimbursement decision-making procedures. For this reason, some stakeholders are rather cautious: “*It is possible that it will become even more complex than it is today*”.

**FIGURE 3:**
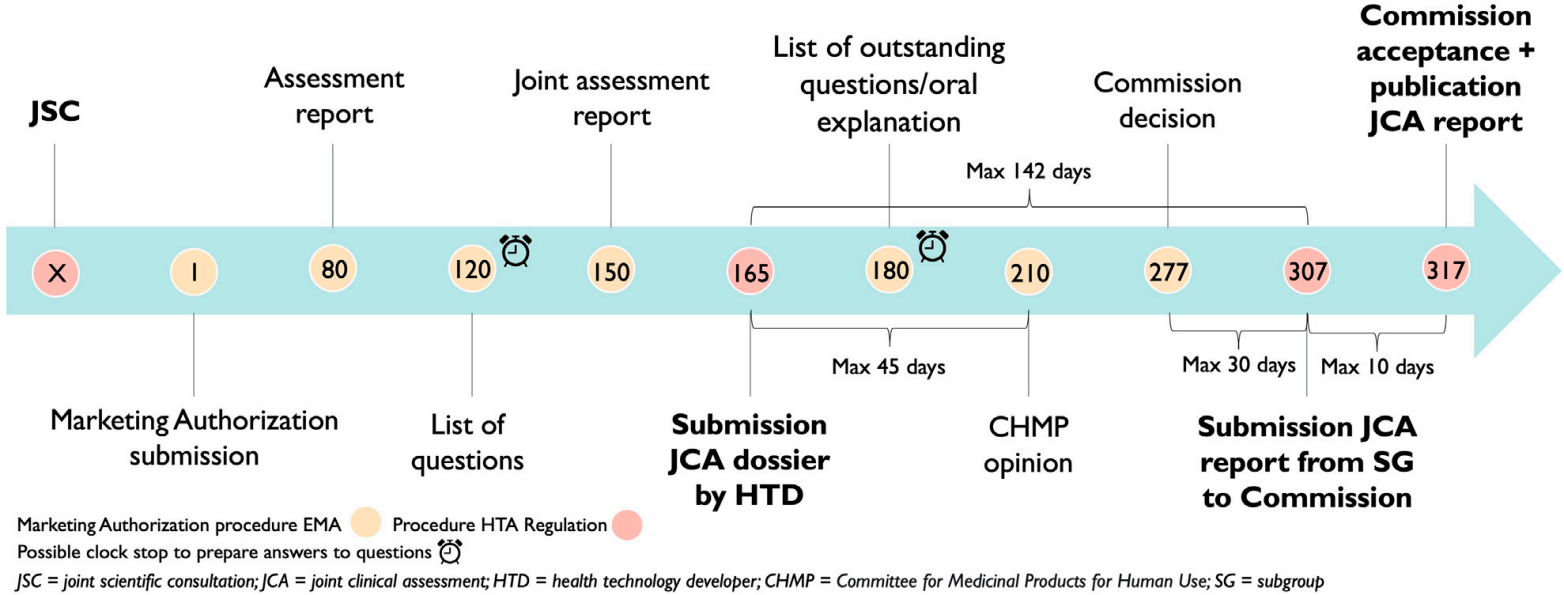
Adoption of the Health Technology Assessment Regulation timelines on the European Medicines Agency timelines.

As described in the HTAR, the timeline above shows the following points in time. The JCA process will start when the HTD has submitted its dossier for a centralized marketing authorization procedure. There is an ultimate timeframe of 142 days (45 days +67 days +30 days) to complete the JCA report. This calculation includes the submission date by the HTD of the JCA dossier which is at the latest 45 days before CHMP opinion, secondly, there is a maximum of 67 days between the CHMP opinion and the Commission’s decision and third, the JCA report will be published no later than 30 days after marketing authorization (Figure 3) (The European Parliament and the Council of the European Union, 2021). These are the ultimate time points since the Coordination Group still needs to: “*adopt detailed procedural steps and the timeframe for the conduct of JCAs and updates thereof”* (The European Parliament and the Council of the European Union, 2021). All stakeholders stressed the importance of not delaying access for patients. To assure this, clearer guidelines are required, in the implementing act, if not as a separate guidance document. Such guidance is now available in the form of the Implementing Act on JCAs of Medicinal Products (European Commissiona; European Commissionb).

Once the first implementing act on the JCA for medicinal products will be published in Q4 of 2023, these aspects will hopefully be cleared out.

### 3.4 Challenges and opportunities

**FIGURE 4:**
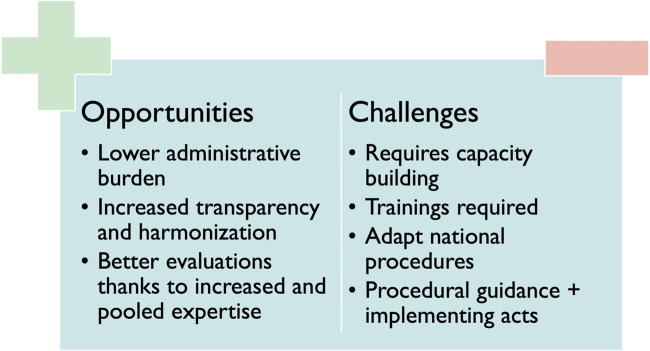
Challenges and opportunities related to HTAR implementation.

The Regulation provides that the (co-)assessors involved in the JSC will be different from the ones involved in the JCA. A stakeholder explained that the (co-)assessors of the JCA will need to take into consideration the advice of the JSC. Interviewees speculated that the reason for this is to avoid conflicts of interest. Some interviewees stated that this could become challenging, especially in fast-emerging therapeutic fields, where the number of experts may be scarce. An overview of the named challenges and opportunities is provided in Figure 4.

#### 3.4.1 Opportunities/benefits

“It’s a learning by doing.”

The primary opportunities and benefits for patients, as identified in the interviews, are associated with clearer and more transparent assessment procedures and assessments. It is expected that these assessments will be of higher quality as expertise is pooled. This could in turn lead to broader access across Europe, bearing in mind that the appraisal, which remains a MS’s authority, follows promptly. Some stakeholders stated that faster access could be linked to the fact that in some MS the procedure of reimbursement could start earlier. Taking into consideration that once the JCA is published, MS could start immediately adopting the JCA in their national dossier requirements and add cost-effectiveness if desired.

*“Ultimately, you know, each country is still going to make its own decision, whether they want to reimburse something. So, I do not think you’re going to see it completely levelled out everywhere. But I think we’re really going to see a shift in it.* (i.e., in the access)*”*

Various stakeholders emphasized the synergetic effects of pooling expertise as the main advantage for HTA bodies compared to the current situation. Moreover, sharing the workload and avoiding duplication were mentioned as opportunities within the HTAR which could improve efficiency.

Regarding HTDs, this gain in efficiency was considered the main benefit due to a reduction in administrative burden as they will have to submit the clinical file only at the European level instead of at 27 different MS. One stakeholder also mentioned that due to the clearer and more harmonized procedures, manufacturers could set expectations for their product earlier as uncertainties on comparative therapy or endpoints can be discussed at the same time as the study design. To ensure this, stakeholders suggested that these guidelines should contain clear criteria on the use of single-arm trials, indirect comparisons and under which circumstances these trials could be conducted.

#### 3.4.2 Challenges

A potential delay in access was mentioned as the main challenge impacting patients as the HTAR could have an impact on (national) timelines of reimbursement procedures resulting in a possibly delayed access.

Capacity building and cooperation with other HTA bodies were mentioned as the main challenges by most interviewees. One interviewee quoted: “*For me, that’s the Achilles heel—finding writers, authors.*” Time pressure to finish all the documents in addition to a suitable remuneration for (co-) assessors remains to be tackled according to most interviewees.

A worry concerning HTDs was about the influence of certain MS on the final JCA report: *“It may be that it is going to be driven by the conservative countries which are possibly the most prominent now in the HTA assessment. Therefore, they will even have less possibility to market their product.”* On top of that, various stakeholders identified internal teamwork as a potential issue, since better internal communication within the different teams of HTDs (e.g., Market Access, Regulatory, Clinical development team) will be required.

#### 3.4.3 Implications on the Belgian national timeline for reimbursement

##### 3.4.3.1 Impact on the Belgian reimbursement procedure

**FIGURE 5:**
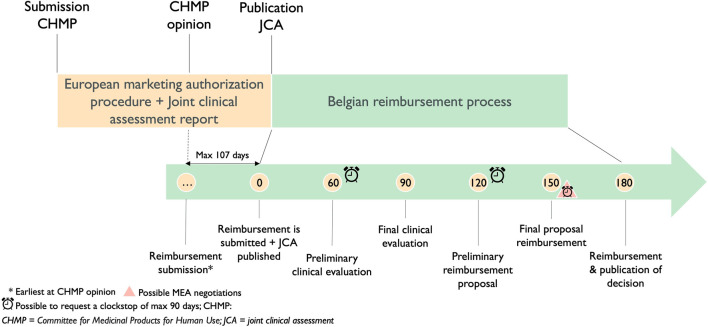
Timeline of the current reimbursement procedure in Belgium in relation to the Joint Clinical Assessment process.

According to the European Transparency Directive (European Communities, 1988), the reimbursement procedure should not exceed 180 days. The current view of several Belgian stakeholders is that this procedure will only start at the time the JCA will be published. Bearing in mind that currently companies can submit for reimbursement at CHMP opinion and publication of the JCA 30 days after marketing authorization, there is a possible delay of 107 days in access for patients in Belgium, as shown in Figure 5.

The timeline for the Belgian procedure below shows the importance of the detailed procedural steps and the timeframe of the conduct of JCAs. Nevertheless, the implementing act on JCA for medicinal products still needs to be published. The opportunity remains to adapt the timeframe of the conduct of the JCA and its detailed procedural steps which could be different, consequently, the start of the reimbursement procedure does not have to be delayed.

##### 3.4.3.2 Actions taken by the Belgian government considering the renewal of the Belgian reimbursement procedure

“*What we think is that Belgium needs to position itself against the EU HTA to play a real active role, otherwise there will be more disadvantages than advantages.”*

With this statement, the stakeholder was referring to the fact that Belgium should decide on its level of involvement—whether active or passive—in the EU HTA process. An active role would mean participating in the preparation phase, and appointing the assessors for JSCs and JCAs so they can have an impact on the way of working by focusing on the important points Belgium values. Conversely, a passive role means Belgium does not actively participate in the preparation of the EU HTA dossiers. Belgium then has the option to either accept or adapt the dossier, providing they have taken it into due consideration and can provide reasoning for non-adoption of the dossier, e.g., not within the Belgian scope or requiring further assessment with local data.

Most (Belgian) stakeholders agreed on the fact that Belgium should play an active role in this process around the implementation of the HTAR. Some stakeholders referred to the fact that Belgium will take the presidency of the Council of the European Union for 6 months from 1 January 2024, onwards. This could also serve as an opportunity to be a pioneer in including the HTAR in their legislation. The Minister of Public Health and Social Affairs is working together with the National Institute for Health and Disability Insurance (NIHDI) on a reform of the reimbursement procedure, which will also take into account the new procedures of the HTAR. A roadmap to renew the reimbursement procedure in Belgium was proposed on the 29th of March 2023 (RIZIV, 2023a; RIZIV, 2023b; RIZIV, 2023c). This roadmap describes broader changes in the reimbursement procedure but specifically in regard to the HTAR, several proposed reforms consider: i) integrating a legal basis to introduce existing (parts of) HTA reports, ii) integrating more elements into the HTA report, iii) considering the JCA properly, whereby the clock of 180 days will only start at the moment of “complete submission dossier”, iv) generalizing the possibility to submit the reimbursement dossier at positive CHMP opinion, v) organizing a Belgian procedure created by NIHDI with the help of the Federal Agency of Medicinal and Health Products, the Belgian Healthcare Knowledge Center and the Commission for Reimbursement of Medicines, to assure the Belgian voice in the JCA procedure, and vi) researching the possibility to exchange/collaborate on the PICO with other countries and afterwards joint negotiations.

##### 3.4.3.3 Beneluxa

Most interviewees agreed on the fact that the future of Beneluxa will be different once the HTAR and its methodologies are put in place. Some stakeholders highlighted the opportunity to (radically) change Beneluxa and its procedures in accordance with the European and updated national procedures. One interviewee emphasized the need for a legal Beneluxa framework. Others underlined a Beneluxa procedure together with a JCA will be in some way duplicate work. The suggestion of compiling a ‘Beneluxa PICO’ was welcomed positively by several stakeholders as it can potentially simplify the consolidation process in preparation for the JSC. The roadmap to renew the Belgian reimbursement procedure provides the investigation of this possibility as well as the acceptance of Beneluxa-assessed medicinal products.

## 4 Discussion

This study has explored stakeholder opinions about the implementation of the European HTAR. Most interviewees are positive about the upcoming changes, yet the implementing acts and procedural guidance will be key to make the JSC and JCA work as envisioned. Furthermore, legal as well as changes in the national reimbursement procedures of MS will need to happen urgently. Recommendations based on the main topics emerging from the interviews are provided.

### 4.1 Consolidation of the PICO

The consolidation of the PICOs is arguably the most challenging concept introduced by the HTAR. This was not only voiced by most of the stakeholders, but it is also often mentioned in public opinions and grey literature (van Engen et al., 2022; Natz, 2023; Schweitzer et al., 2023). Several interviewees shared their disquiets about the expected number of consolidated PICOs. Historically, MS have employed diverse criteria for healthcare decision making. Besides, the number of alternative treatments available can differ across various countries (Drummond et al.). It is therefore important to have training and guidelines to foresee that every MS will construct its individual PICO by the same method to improve the consolidation process. Julian et al. recognized four domains that should be addressed in the guidance and/or implementing acts: i) the process, ii) the uncertainty, iii) the comparator choice, and iv) the endpoint selection (Julian et al., 2022a). In that spirit, Drummond et al. recommended having standards for i) the selection of comparators, ii) analysis of the data and iii) the role of synthetic patient cohorts and *in silico* modelling (Drummond et al.).

These concerns were not unfounded as the EUnetHTA consortium has tested their protocols on three EMA-approved medicinal products. The deliverable “JCA without HTD submission” showed that when (respectively) eight, ten and ten MS completed the survey for lutetium (^177^Lu) vipivotide tetraxetan (Pluvicto^®^), tabelecleucel (Ebvallo^®^) and cipaglucosidase alfa (Pombiliti^®^), this resulted in respectively six, five and nine consolidated PICOs (EUnetHTA, 2023a; EUnetHTA, 2023b; EUnetHTA, 2023c; EUnetHTA, 2023d). It can thus be expected that when all 27 MS are involved this could become even more complicated. Van Engen et al. analyzed the impact of additive PICOs in lung cancer and revealed an expectation of up to ten different PICOs within one submission (van Engen et al., 2022). In this regard, the European Confederation of Pharmaceutical Entrepreneurs (EUCOPE) and the European Federation of Pharmaceutical Industries and Associations (EFPIA) have taken the position that to harness the benefit of a “one-stop-shop”, the number of comparators should be limited to three for any given JCA (EUCOPE EFPIA., 2022). It is thus very welcomed that the EUnetHTA consortium has advised to set up a working group to optimize this consolidating procedure (EUnetHTA, 2023d).

A first recommendation to the European Commission is to provide clear assistance in defining the steps needed to compile a PICO in a standardized manner by every MS.

### 4.2 Early dialogue and the joint scientific consultation

Since the JSC is a voluntary process, interviewees stressed the importance of estimating the number of health technologies that could benefit from JSC. Through such a horizon scan, the subgroup for identification of emerging health technologies was given a crucial responsibility to foresee enough assessors and co-assessors for every JSC or JCA to be conducted and thus prevent being faced with a lack of capacity.

Next to the fear of being faced with a lack of capacity, there is a concern about duplicate work at the regulatory and HTA levels. It is well known that there is heterogeneity between regulatory and HTA evidence requirements (Julian et al., 2022b). Julian et al. therefore identified four value drivers that need focus in HTAs nowadays: i) small patient populations, ii) innovative study designs, iii) RWD sources and, iv) new endpoints (Julian et al., 2022b). This advocates, as stated by EFPIA and EUCOPE to offer early dialogue to the HTDs that seek advice from regulators and HTA agencies in order to deliver timely JCAs (EUCOPE EFPIA., 2022). To these matters, Schweitzer et al. suggested including a mandatory and early JSC for meaningful interactions with the HTDs. These early engagements would aid in the incorporation of consolidated PICOs in the study planning of HTDs. They believe that carefully chosen dossier requirements, compulsory JSCs and HTD involvement can minimize redundant efforts for both HTA bodies and HTDs (Schweitzer et al., 2023). It is henceforth proposed to design the guidelines and implementing acts while using the principles of change management (Raza and Standing, 2011). This could result in more engagement of the pharmaceutical industry as their needs can also be addressed which is lacking now according to several interviewees.

### 4.3 Joint clinical assessments: an obligation to take into consideration

“*JCA can be a quantum leap for market access in the EU.*” (Gianfrate, 2023)

Although the interviewees recognized the value of the HTAR and the acceptance of the JCA reports in national decision making is anticipated to be high (Natz, 2023), it does not have any legal or binding force in terms of deciding on reimbursement for MS. Some might get the impression that HTA bodies will deviate from the JCA report and request additional evidence or complementary clinical analysis if not yet requested by other MS. Because of this, the HTAR could have a wide-reaching indirect effect on price and reimbursement because countries could be tempted to cherry pick only the preferred outcomes from the PICO menu. This would paradoxically increase the heterogeneity between MS, exactly the opposite of the harmonization goal of the HTAR initiative (Gianfrate, 2023). Conversely, it could also result in a spill-over effect, where the more conservative HTA approaches and agencies influence the more pragmatic and less dogmatic ones. Next to this, the HTAR is expected to impact the national timelines, which could have strategic implications for HTDs. It is therefore recommended to provide clear guidelines to increase homogeneity in writing JCA reports as well as install training to prevent any misinterpretation by MS when adopting the EU HTA report.

### 4.4 Challenging methodological and procedural guidance on JCA

The implementing acts and guidelines on the procedural steps and timeframe to conduct a JSC and JCA remain to be determined by the coordination group. These guidelines and implementing acts must build on transparency and rely on evidence-based methodologies (Hwang and Vokinger, 2022), without introducing additional layers of complexity or additional barriers (EUCOPE EFPIA., 2022). The implementing act on JCA for medicinal products will be the first one to be published. In the fifth meeting of the Member State Coordination Group, the Commission informed the members that the draft text would be shared with the HTA Committee in advance of the next meeting mid-November 2023 (Directorate-General for Health and Food Safety, 2023b). However, the flash report of the sixth meeting omitted any mention of this draft text (Directorate-General for Health en Food Safety, 2023), suggesting a potential delay in its publication. In the February 2024 update of the Implementation Rolling Plan, this postponement to Q1 2024 was confirmed (European Commissionc). Finally, on March 5, the long-awaited public consultation on the draft Implementing Act was published by the European Commission (European Commissiona; European Commissionb).

Interviewees stressed the need for proper guidelines to allocate (co-)assessors with the right expertise regarding different health technologies, e.g., complex gene therapies, new blockbusters, me-too products, and others. This aspect was also highlighted by EUCOPE in its priory list stating “*A transparent and balanced selection of experts for rare diseases and specialized technologies is necessary*” (EUCOPE, 2022a).

Finally, based on the analysis of the interviews it is consequently advised to translate the JSC and JCA reports to the various national languages to facilitate the adoption to the local reimbursement procedures.

### 4.5 Patient access should not be delayed

The proposed timelines by the EUnetHTA21 consortium are quite strict. EUnetHTA21’s deliverable 4.2 (EUnetHTA, 2023e) on the scoping procedure of the PICO envisages approximately 2 weeks. After conducting their three PICO exercises EUnetHTA21 (Pluvicto^®^, Ebvallo^®^ en Pombiliti^®^) concluded these timelines might be too strict (EUnetHTA, 2023a; EUnetHTA, 2023b; EUnetHTA, 2023c; EUnetHTA, 2023d; Willemsen, 2023).

The JCA will be the same for all MS, yet the usage can differ in decision making across MS. Furthermore, one might think that although the JCA will only cover the clinical assessment, it might have an indirect effect on price and reimbursement negotiations as for example, comparators and endpoints will be defined at a European level during the PICO consolidation process (Natz, 2023).

Schweitzer et al conducted a study on a suitable EU HTA dossier template concluding current information strongly indicates that the draft submission dossier template and submission dossier are based on the highly rigorous German Arzneimittelmarkt-Neuordnungsgesetz (AMNOG) requirements. These requirements are extensive as writing the AMNOG dossier takes approximately 12 months. The proposed timelines in the HTAR are 2.5 months. These disparate timelines could pose a significant risk for patient accessibility which might result in delays (Schweitzer et al., 2023).

As mentioned before, to avoid any delay in access for patients, MS must adapt their reimbursement procedures and timelines in accordance with the HTAR.

### 4.6 Taking collaborative work one step further

As evaluated positively by several interviewees, the Beneluxa members could compile a joint Beneluxa PICO, which would represent their joint interests and in turn could give more weight to these small MS during the consolidation procedure. The Beneluxa initiative has already proven to be effective in ensuring sustainable access to innovative therapies, i.e., the case of Zolgensma^®^. The Belgian Minister of Social Affairs and Public Health has described the Beneluxa Initiative as *“… a strong ‘brand’ and gold standard for voluntary collaboration between member states.*” (BeNeLuxA, 2018). This exemplifies the strength of existing collaboration between these MS, and advocates for further collaboration in the light of the HTAR. Therefore, Beneluxa could take an example from the FINOSE collaboration (Norwegian Medicines Agency, 2023) which is already investigating how to accommodate and prepare for the requirements of the HTAR.

### 4.7 Limitations

Given that only very few articles have been written so far on the impact and implementation of the HTAR, the peer-reviewed literature on the subject was limited. Moreover, at present, no drafts of the crucial implementing acts or of the national legislations adapting the national framework have been made available. However, EUnetHTA21’s deliverables give a direction. Something that was put forward by the heads of the HTA agencies group during the sixth meeting of the Member State Coordination Group was that they “*requested clarity on the funding of the joint work under the HTAR*” (Directorate-General for Health en Food Safety, 2023). Although this confirms the concern voiced during the interviews, further investigation is needed to thoroughly examine this aspect.

Secondly, nevertheless, we believe that the selected five different stakeholder groups represented the most concerning parties and saturation was reached, it is always possible to miss out on crucial insights.

Lastly, it is worth noting that although this study only focussed on the HTA processes, differences in budgets are likely a great contributor to the inequality of patient access across MS.

### 4.8 Future research

One of the EUnetHTA21’s last deliverables regarded a collaboration with the EMA during JSCs. This could be an efficient collaboration and further research on it should be conducted.

The impact of the upcoming changes introduced by the new pharmaceutical legislation should be considered during the drafting of the implementing acts and the adaptations of the national reimbursement procedures. During the sixth meeting of the coordination group, several members had questions regarding access to medicines, market access, pricing, and comparative clinical trials for both HTA and marketing authorization.

Furthermore, once the implementing acts are finished and the timeframes of the procedures are decided, the integration of these guidelines into the national reimbursement procedures for every MS will be interesting to follow. Moreover, the impact on the timelines for patients on access should be evaluated.

## 5 Conclusion

Our study on the implementation of the European HTAR has indicated that the success or failure of these newly introduced concepts (JSC and JCA) lies in the implementing acts, procedural guidance/timelines, and guidelines. It is important to keep in mind that the ultimate goal of the HTAR is to gain efficiency, lower administrative burden, and improve and equalize access to all patients across Europe. Our study also concluded that only in case the procedures are convenient for both payers/HTA bodies and HTDs, the procedures introduced by the HTAR will be successful. Additionally, there is space for other collaborative work between MS such as Beneluxa and FINOSE. Furthermore, MSs should start preparing for implementation and adapt their legislation where necessary. Lastly, clear guidelines on the quality of data, clinical trial design, comparators and endpoints must be provided as soon as possible.

## Data Availability

The raw data supporting the conclusion of this article will be made available by the authors, without undue reservation.
