# Supplementary material for: Implementing the EU HTA regulation: Insights from semi-structured interviews on patient expectations, Belgian and European institutional perspectives, and industry outlooks
Source: Front Pharmacol. 2024 Apr 10;15:1369508. doi: 10.3389/fphar.2024.1369508 (PMC11039851; doi:10.3389/fphar.2024.1369508)
Supplement: Supplementary file 1 [file DataSheet1.docx]

Supplementary Material

Implementing the EU HTA Regulation: Insights from semi-structured interviews on patient expectations, Belgian and European institutional perspectives, and industry outlooks.

Thomas Desmet^†, *^, Maud Brijs^†^, Frank Vanderdonck, Sven Tops, Steven Simoens, Isabelle Huys

**† These authors share first authorship**

*** Correspondence:** Thomas Desmet - [Thomas.desmet@kuleuven.be](mailto:Thomas.desmet@kuleuven.be)

# Supplementary Data

**Questions for stakeholder group 1: Belgian governmental instances**

I. Vragen over het thema "HTA's"

1. Wat zijn volgens u **lacunes** in het huidige Belgische HTA-kader dat gehanteerd wordt in de beoordeling van een terugbetaling? (probeer er minstens 3 uit te halen)
2. Wat is uw mening over de terugbetaling van ATMP's in het huidige systeem in België?
   1. Wat zijn volgens u optimaliseringsmaatregelen om het huidige systeem beter af te stemmen op de evaluatie van ATMP's?
3. Welke **criteria** worden in België gebruikt voor de **beoordeling** van de klinische en economische waarde van ATMPs?
4. Wat zijn voor u de **data gaps** die maken dat een klinische beoordeling moeilijk maakt.
   1. Hoe gaat u om met deze **onzekerheden** in uw beoordeling?
5. Hoe moeten **bedrijven** volgens u omgaan met deze **onzekerheden**?
   1. Subvraag 1: Hoe moet er volgens u omgegaan worden met het gegeven dat in de pivotale studies een comparator ontbreekt of niet overeenkomt met de Belgische praktijk?
   2. Subvraag 2: Wat vindt u van de toevoeging van een “**synthetic comparator**” op basis van Belgische historische patiënten data?
   3. Subvraag 3: Hoe staat u tegenover indirecte vergelijkingen, MAICs (matching-adjusted indirect comparisons), als alternatief/oplossing voor het gebrek van een comparator.
   4. Subvraag 4: Welke andere oplossingen/voorstellen zijn er voor het aanpakken van data tekorten?
   5. **Subvraag 5:** Is **vooroverleg** nodig mogelijk/nuttig om data gaps te benoemen en op voorhand afspraken te maken over hoe dit aangepakt dient te worden. Indien ja, hoe? Indien neen, waarom niet?
6. Hoe staat u tegenover de mogelijkheid om een **voorwaardelijke terugbetaling** in te voeren na het aanleveren van nieuwe of RWE data?
   1. Bijvoorbeeld als bepaalde doeltreffendheid of eindpunten worden bereikt volgt een automatische een definitieve inschrijving? Zijn er tussenopties mogelijk?

II. Vragen over het thema "European joint clinical assessment "JCA""

1. Wat is uw mening over de invoering van de JCA?
2. Wat zullen volgens u de grootste **voordelen** van de JCA zijn voor:
   1. HTA organen van de lidstaten in het algemeen en België in het bijzonder?
   2. De farmaceutische bedrijven die ATMP’s ontwikkelen?
   3. Patiënten?
3. Wat zullen volgens u **uitdagingen** van de JCA zijn voor:
   1. HTA organen van de lidstaten in het algemeen en België in het bijzonder?
   2. De farmaceutische bedrijven die ATMP’s ontwikkelen?
   3. Patiënten?
4. Wat vindt u van de invoering van de **PICO** (Patient/Intervention/Comparator/Outcomes)?
   1. Wat zijn volgens u voordelen van de introductie van de PICO?
   2. Zijn er volgens u ook nadelen? Zo ja, welke?
   3. EXTRA: Denkt u dat implementatie van een PICO kan bijdragen tot meer uniformisering en standaardisering van het proces?
   4. Welk orgaan in België is er volgens u het best geschikt om een PICO te bepalen voor België ‘op het moment van de joint scientific consultation?
      1. Is dit een taak enkel weggelegd voor het RIZIV of zou het KCE hier ook zijn expertise ter beschikking kunnen stellen?
5. Wat vindt u van het idee om het scientific advise van EMA samen met de JSC te laten plaatsvinden?
6. Wat vindt u van het feit dat de assessors voor de JSC en de assessors voor de JCA verschillend moeten zijn?
7. Welke **rol** kan/zal **België** volgens u spelen tijdens het proces van de gezamenlijke klinische evaluatie?
   1. Wie is er voor België verantwoordelijk in de coordination group?
      1. Hoe ziet u de rol van het KCE hierin?
   2. Wordt er voldoende capaciteit voorzien om actief deel te nemen aan zowel de JSC als de JCA?
   3. Welk orgaan gaat zich bezighouden met het nakijken van de methodologie en het vertalen naar de nationale situatie?
8. Hoe zal de JCA gebruikt worden in het **Belgische klinische beoordelingsproces**?
   1. Subvraag 1: In welke mate ziet u de noodzaak van een herbeoordeling door de interne deskundigen van het RIZIV?
      1. Zo ja, wat zou volgens u een legitieme reden kunnen zijn voor een herevaluatie?
      2. Kunt u de meerwaarde van een eventuele herevaluatie aangeven?
      3. Kunt u ingaan op de nadelen van een herevaluatie?
   2. Subvraag 2: Is er een tussentijdse mogelijkheid om bijvoorbeeld alleen een herevaluatie te doen als de comparator in de JCA anders is dan de Belgische?
      1. Is er dan een mogelijkheid dat dit op het moment dat de PICO bepaald wordt dus de JSC dat er dan al op nationaal niveau kan gezegd worden van voor ons ontbreekt dit dus als jullie naar België willen komen is dit ook nog nodig om een volledige evaluatie te kunnen voeren?
   3. Welke aanpassingen zijn volgens u nodig om de JCA te integreren in de Belgische terugbetalingsprocedure? Moet er iets fundamenteel veranderen aan de Belgische terugbetalingsprocedure? Zo ja, wat moet er veranderd worden?
      1. Kunt u ingaan op de uitdagingen van deze aanpassingen?
      2. Welke praktische gevolgen zullen deze veranderingen hebben voor het functioneren van de CTG?
      3. Vindt u dat er **2 verschillende comité’s** moeten opgericht worden? Bijvoorbeeld eentje die zich vooral bezig houdt met het klinische deel en bijvoorbeeld ook engageert om deel te nemen aan de JSC en JCA’s en de andere die vooral de economische evaluatie/BI analyseert?
   4. Hoe zou België ervoor kunnen zorgen dat de markttoegang niet vertraagd wordt voor de patiënten? Gaat de periode van 180 dagen ingekort worden? Of gaat deze pas starten op de dag dat de JCA is gepubliceerd (max 30d na CHMP opinie)? Dit zal namelijk zorgen voor een vertraging in de toegang voor de patiënten?
   5. Heb ik het goed begrepen dat het idee is dat de tijdslijn in België gaat opgeschoven worden met max 30 dagen, namelijk totdat het terugbetalingsdossier volledig is?
   6. Gedurende het volledige proces van terugbetaling, dus vanaf de early dialogues en JSC, vindt u dat er een betere samenwerking moet komen tussen het KCE en het RIZIV? Er zit namelijk ook veel specifieke expertise bij het KCE en misschien zijn beide complementair?
      1. Vindt u het KCE het geschikte orgaan om de experten van het RIZIV te voorzien van een verdere vormgeving aan de hand van extra trainingen specifiek voor bepaalde groepen van geneesmiddelen bijvoorbeeld ATMP’s?
      2. Maar ook trainingen in hoe een goede HTA uitgevoerd zou moeten worden?
   7. Ik las in de synthese tabel van het voorstel voor de hervorming van de CTG dat er gemikt wordt op een aanpassing of vernieuwing van de wet midden 2023. Wat is nu de status van het wetgevend proces?
      1. Is er al een voorontwerp van wet gemaakt?
9. In artikel 13 van de regulation staat beschreven wat de rechten en plichten zijn van de lidstaten; elke lidstaat moet de JCA bij zijn terugbetalingsdossier voegen, hiermee dus rekening houden en vervolgens binnen de 30 dagen meedelen aan de EC op welke manier zij die bepaalde JCA in aanmerking hebben genomen. Weliswaar staan daar geen sancties op staan, maar hoe gaat België hiermee omgaan?
   1. Wie gaat hiervoor instaan om dit tijdig op het IT platform te registreren?
10. Wat is de impact van JCA op het **BENELUXA** initiatief?
    1. Ziet u een oportuniteit voor dit initiatief?
    2. Er zou bijvoorbeeld een PICO op dit niveau samengesteld kunnen worden?
    3. Acht u het nodig om voor Beneluxa een aparte procedure te voorzien zodat de beslissing die wordt genomen op beneluxa-niveau rechtstreeks geldt op nationaal niveau in België?
11. Op welke vlakken moet **EUnetHTA** nog bijkomende/cruciale stappen ondernemen om ervoor te zorgen dat de implementatie in 2025 vlot kan gebeuren?
    1. Welke problemen verwacht u bij het implementeren van de JCA?
    2. Hoe denkt u dat deze problemen dienen aangepast te worden?
12. Extra: Vindt u dat naast de klinische beoordeling er ook een Europese overeenkomst moet gezocht worden naar de graad van medical need, interpretatie van een bepaalde impact op een gezondheidsuitkomst (bv erg klinisch relevant, klinisch relevant, minder klinisch relevant, niet relevant)?
13. Wat is het standpunt van u, uw organisatie van de Belgische overheid over de toegevoegde waarde/nut van JCA?
14. Welke stappen moeten er volgens u nog ondernomen worden vooraleer België echt klaar is om de JCA te implementeren in het terugbetalingssysteem in 2025?
15. EXTRA: Wat zijn de gevolgen van JCA voor de gelijke toegang tot patiënten in alle lidstaten?
16. EXTRA: Wie zou volgens u allemaal moeten **betrokken** worden in de keuze om de vergelijkende therapie te kiezen?
17. EXTRA: Wat is uw opinie over de invoering van een Europese JCA, eerst voor ATMP’s en oncologische geneesmiddelen, daarna voor weesgeneesmiddelen en uiteindelijk voor alle geneesmiddelen?

III. Vragen over het thema "ATMP's"

1. Wat zijn volgens u de **belangrijkste uitdagingen**, nu en in de toekomst bij het evalueren van ATMP's?
2. EXTRA: Denkt u dat een JCA een **oplossing** kan/zal bieden voor deze uitdagingen?
3. Hoe kan er met een **beperkte patiëntenpopulatie** toch een betrouwbaar resultaat omtrent effectiviteit en veiligheid besloten worden?
   1. Subvraag 1: Op welke manier zal de JCA hierop effect hebben?
   2. **Subvraag 2:** Denkt u ook dat **verdere Europese samenwerking** nodig is: Bijvoorbeeld bij de collectie/analyse van RWD?
      1. **Wie moet volgens u dan allemaal deel uit maken van deze samenwerking?**
   3. **Vindt u dat er meer gebruik gemaakt moet worden van patient-reported-outcomes?**
   4. Subvraag 3: Hoe kunnen bedrijven zicht hierop voorbereiden?
   5. Subvraag 4: Dient dit in overleg te gaan met de overheden?
   6. Subvraag 5: Denkt/verwacht u dat JCA een invloed kunnen hebben op het verdere proces van nieuwe methoden die kunnen gebruikt worden data gaps op te lossen?
4. Welke waarde hecht u aan **lange termijn RWE data** in de (her)beoordeling van een terugbetaling?
   1. Bent u een voorstander
5. EXTRA: Denkt u dat JCA het proces van **toegang** tot ATMPs gaat **versnellen**? Waarom?

I. Questions on the topic of "HTAs"

1. What do you think are **gaps** in the current Belgian HTA framework used in reimbursement procedure of ATMPs? (try to get at least 3 out)
2. What is your opinion on the reimbursement of ATMPs in the current system in Belgium?
   1. What do you think are optimisation measures to better align the current system with the evaluation of ATMPs?
3. What criteria are used in Belgium to assess the clinical and economic value of ATMPs?
4. What are the **data gaps** that make a clinical assessment difficult for you?
   1. How do you deal with these uncertainties in your assessment?
5. How do you think **companies** should deal with these **uncertainties**?
   1. How do you think the fact that pivotal studies lack a comparator or does not correspond to Belgian practice should be dealt with?
   2. What do you think of the addition of a "synthetic comparator" based on Belgian historical patient data?
   3. How do you feel about indirect comparisons, MAICs (matching-adjusted indirect comparisons), as an alternative/solution to the lack of a comparator?
   4. What other solutions/proposals are there for addressing data shortages?
   5. Is preliminary consultation possible/useful to identify data gaps and make agreements in advance about how this should be handled. If so, how? If not, why not?
6. How do you feel about the possibility of introducing a **conditional reimbursement** after the collection of new or RWE data?
   1. For example, if certain effectiveness or endpoints are reached, does an automatic final enrollment follow? Are there intermediate options?

II. Questions on the theme "European joint clinical assessment "JCA""

1. What is your opinion on the introduction of the JCA?
2. What do you think the biggest **benefits** of the JCA will be for:
   1. HTA bodies of the Member States in general and Belgium in particular?
   2. The pharmaceutical companies that develop ATMPs?
   3. Patients?
3. What do you think the JCA's **challenges** will be for:
   1. HTA bodies of the Member States in general and Belgium in particular?
   2. The pharmaceutical companies that develop ATMPs?
   3. Patients?
4. What do you think of the introduction of the **PICO** (Patient/Intervention/Comparator/Outcomes)?
   1. What do you think are the benefits of introducing the PICO?
   2. Do you think there are any disadvantages? If so, which ones?
   3. EXTRA: Do you think that implementation of a PICO can contribute to more uniformization and standardization of the process?
5. What **role** do you think **Belgium** can/will play in the joint clinical assessment process?
6. How will the JCA be used in the **Belgian clinical assessment process**?
   1. To what extent do you see the need for a reassessment by the in-house experts of the NIHDI?
      1. If so, what do you think could be a legitimate reason for a reevaluation?
      2. Can you indicate the added value of a possible re-evaluation?
      3. Can you elaborate on the disadvantages of a reassessment?
   2. Is there an interim possibility to only do a reassessment, for example, if the comparator in the JCA is different from the Belgian one?
   3. What adjustments do you think are needed to integrate the JCA into the Belgian reimbursement procedure? Does something fundamentally need to change about the Belgian reimbursement procedure? If so, what needs to be changed?
      1. Can you address the challenges of these adjustments?
      2. What practical consequences will these changes have for the functioning of the CTG?
      3. Do you think **2 different committees** should be set up? For example, one that is mainly concerned with the clinical part and, for example, also commits to participate in the JSC and JCAs and the other that mainly analyzes the economic evaluation / BI?
   4. How could Belgium ensure that **market access is not delayed for patients**? Will the 180-day period be shortened? Or will it only start on the day the JCA is published (max 30d after CHMP opinion)?
7. In what areas does **EUnetHTA** need to take additional/crucial steps to ensure that implementation can be done smoothly in 2025?
   1. What problems do you expect when implementing the JCA?
   2. How do you think these problems need to be addressed?
   3. Why would the EUnetHTA21 consortium be the right organization to give **trainings** on the deliverables to member state and HTA bodies? Or why not?
   4. Which steps are taken to be sure that all the JCA’s will be done in time so that the patient’s access won’t be delayed or hindered?
      1. Is there enough **capacity** provided to assess all the ATMP’s in the pipeline?
   5. **How many advice** will be given in this implementation phase, from 2025 on? What is the scale up and capacity building that you pursue?
   6. Why would the EUnetHTA21 consortium be the right organization to give **trainings** on the deliverables to member state and HTA bodies? Or why not?
   7. Will the assessment be published in other languages besides English or is translation something member states should provide themselves if they need it?
8. What is the impact of JCA on the **BENELUXA** initiative?
9. The regulation states that the **assessor of the JSC and the assessor of the JCA** need to be different, what is your opinion on tis decision? It seems a bit odd to me. What if the assessor of the JCA does not agree with what the assessor of the JSC said?
10. It appears that the JSC will still need to be requested by the HTD like it was the case in the Joint Actions and EUnetHTA21. What can be an advantage to organize it this way?
11. The accessor and co-assessor of the JSC and the JCA need to be different. Why do you think this is the case?
12. Extra: Do you think that in addition to the clinical assessment, a European agreement should also be sought on the degree of medical need, interpretation of a certain impact on a health outcome (e.g. very clinically relevant, clinically relevant, less clinically relevant, irrelevant)?
13. What is your position of you, your organization of the Belgian government on the added value /usefulness of JCA?
14. EXTRA: What are the implications of JCA for equal access to patients in all Member States?
15. EXTRA: Who do you think should be **involved** in the choice to choose comparative therapy?
16. EXTRA: What is your opinion on the introduction of a European JCA, first for ATMPs and oncology medicines, then for orphan drugs and finally for all medicines?

III. Questions on "ATMP's"

1. What do you think are the **most important challenges**, now and in the future, when evaluating ATMPs?
2. EXTRA: Do you think a JCA can/will provide a solution to these challenges?
3. With a **limited patient** and **population,** how can a reliable result in terms of effectiveness and safety be decided?
   1. Sub-question 1: How will the JCA have an effect on this?
   2. Subquestion 3: How can companies prepare for this?
   3. Sub-question 4: Should this be done in consultation with the authorities?
   4. Subquestion 5: Do you think/expect that JCA can have an impact on the further process of solving new methods that can be used to solve data gaps?
4. What value do you attach to **long-term RWE data** in the (re)assessment of a reimbursement?
   1. Do you also think that **further European cooperation** is needed: For example, in the collection/analysis of RWD?
5. EXTRA: Do you think JCA is going to **speed up** the process of **accessing** ATMPs? Why?

**Questions for stakeholder group 2: European agencies and organizations**

I. Vragen over het thema "HTA's"

1. Welke **lacunes** zijn er **in het huidige HTA-kader** voor ATMP's dat door de afzonderlijke lidstaten wordt gebruikt? (Probeer er 3 te bedenken)
2. EXTRA: Wat zijn voor u de hiaten in de gegevens die een **klinische beoordeling** bemoeilijken?
   1. Hoe gaat u in een beoordeling om met deze onzekerheden?
3. Hoe moeten ontwikkelaars omgaan met deze **onzekerheden**?
   1. Subvraag 1: Hoe moet volgens u worden omgegaan met het feit dat in de kernstudies een vergelijkingsfactor ontbreekt of niet overeenkomt met de praktijk van een lidstaat?
   2. Subvraag 2: Wat vindt u van de toevoeging van een "**synthetische comparator**" op basis van de historische patiëntgegevens van een lidstaat?
   3. Subvraag 3: Wat is uw standpunt over indirecte vergelijkingen of matching-adjusted indirecte vergelijkingen (MAIC's) als alternatief/oplossing voor het ontbreken van een comparator?
   4. Subvraag 4: Kunt u een voorbeeld geven van andere oplossingen/voorstellen om datatekorten aan te pakken?
4. EXTRA: Denkt u dat het mogelijk is om een **voorwaardelijke vergoeding** in te voeren na het indienen van nieuwe of RWE-gegevens?
   1. Als bijvoorbeeld bepaalde effectiviteit of eindpunten worden bereikt, kan er dan automatisch een definitieve registratie volgen? Zijn er tussenliggende opties?
   2. In de regulation staat dat er een actualisering mogelijk is op vraag van de leden van de coordinatiegroep, kan dit ook op vraag van de HTD?
5. EXTRA: Hoe ga je om met **onzekerheden** in een assessment?

II. Vragen over het thema "European joint clinical assessment (JCA)"

1. Wat is uw opinie over de invoering van een Europese JCA?
   1. Voor ATMPs en oncologische geneesmiddelen
   2. Voor weesgeneesmiddelen
   3. Voor alle geneesmiddelen
2. Wat zullen volgens u de grootste **voordelen** van de JCA zijn voor:
   1. HTA-agentschappen van de lidstaten
   2. De farmaceutische bedrijven (die ATMP’s ontwikkelen)
   3. Patiënten
3. Wat zullen volgens u **uitdagingen** van de JCA zijn voor:
   1. HTA-agentschappen van de lidstaten
   2. De farmaceutische bedrijven
   3. Patiënten
4. Welke andere opties waren er naast de PICO-evaluatie? Wat maakte dat PICO opviel/verkozen werd boven de andere opties die op dat moment op tafel lagen?
   1. Welke nadelen zou u toewijzen aan het gebruik van de PICO?
   2. Op welke manier denkt u dat de invoering van de PICO kan bijdragen tot meer uniformiteit en standaardisering van het proces?
5. Wat zijn **cruciale stappen die EUnetHTA** nog moet zetten om een soepele implementatie te hebben?
   1. Subvraag 1: Welke problemen verwacht u bij de implementatie?
   2. Subvraag 2: Hoe kunnen deze problemen worden aangepakt?
   3. Subvraag 3: Zal EUnetHTA klaar zijn om de JCA te beoordelen voor alle ATMP's in de pijplijn?
   4. Subvraag 4: Weet u hoeveel adviezen er zullen worden gegeven in 2025?
      1. Wat is de opschaling en capaciteitsopbouw die u doorzet?
   5. Subvraag 5: Welke stappen worden er genomen om er zeker van te zijn dat alle JCA's op tijd worden gedaan, zodat de toegang van de patiënt niet wordt vertraagd of belemmerd?
   6. Subvraag 6: Zullen er vertalingen voorzien worden van de assessment?
6. Wat zijn de belangrijkste **veranderingen** die elke **lidstaat** vóór 2025 moet aanbrengen in zijn evaluatiestrategieën/-kader om voorbereid te zijn op het gebruik van de JCA?
7. Op welke manieren proberen jullie met **EUnetHTA21** de lidstaten zo goed mogelijk te informeren en te **begeleiden** bij de voorbereiding voor 2025?
   1. Gaat EUnetHTA naast de deliverables ook trainingen/webinars organiseren om de HTAb van de lidstaten op de leiden om bijvoorbeeld een goede PICO op te stellen, hoe de JCA opgesteld moet worden, mogelijke implementatie, …
   2. Zal er hulp voorzien worden voor de lidstaten om hun capaciteit te verhogen om de dossiers mee te kunnen schrijven en voldoende grondig te kunnen analyseren?
8. EUnetHTA21 heeft een contract tot september 2023 maar wie gaat daarna de organisatie voor zich nemen? Zal er een nieuwe **tussentijdse werkgroep** komen of wordt op dat moment de **coördinatiegroep** ingesteld?
9. Denkt u dat de JCA **het proces van toegang tot ATMP’s gaat versnellen**? Waarom?
10. Vindt u dat naast de klinische beoordeling er ook een Europese overeenkomst moet gezocht worden naar de graad van medical need, interpretatie van een bepaalde impact op een gezondheidsuitkomst (bv erg klinisch relevant, klinisch relevant, minder klinisch relevant, niet relevant)?
11. Wat is de impact van de JCA op het **BENELUXA-initiatief** of andere cross-country collaborations?
12. Het idee is dat er een eerdere/vroegtijdige en meer **inclusieve samenwerking** komt tussen de verschillende actoren, maar hoe wordt dat geïmplementeerd? In welke stadia/hoe vaak/hoe te overwegen/...?
13. EXTRA: Waarom heeft de Europese Commissie ervoor gekozen **om de volgorde van implementatie** te kiezen uit ATMP's en oncologische producten (in implementatiefase), weesgeneesmiddelen (vanaf 2028) en ten slotte alle geneesmiddelen?
14. EXTRA: Hoe gaat een verschil in implementatie van de JCA door de verschillende lidstaten (op vlak van prijsbepaling, terugbetaling etc.) geminimaliseerd worden?
    1. Hoe gaan jullie omgaan met het feit dat er in sommige landen misschien regelmatig extra data wordt gevraagd?
15. EXTRA: Denkt u dat er, ondanks de JCA, **heterogeniteit** tussen de lidstaten zal blijven bestaan wat betreft de gevolgen voor de toegang voor patiënten?
16. EXTRA: Hoe zal Europa ervoor zorgen dat de JCA **door elke lidstaat wordt geïmplementeerd/gebruikt**?

III. Vragen over het thema "ATMP's"

1. Wat zijn **belangrijke uitdagingen**, nu en in de toekomst, van het evalueren van ATMP's?
2. EXTRA: Denkt u dat de JCA in staat zal zijn om **deze uitdagingen aan te pakken**?
3. Hoe kan een betrouwbaar resultaat met betrekking tot effectiviteit en veiligheid worden bepaald met een **beperkte patiëntenpopulatie?**
   1. Subvraag 1: Hoe zal de JCA hierop van invloed zijn?
   2. Subvraag 2: Denkt u ook dat verdere Europese samenwerking nodig is: bijvoorbeeld bij het verzamelen/analyseren van patiëntgegevens uit het echte leven/RWE?
   3. Subvraag 3: Hoe kunnen bedrijven zich hierop voorbereiden?
   4. Subvraag 4: Moet dit met de autoriteiten worden besproken?
   5. Subvraag 5: Denkt/verwacht u dat JCA een impact kan hebben op het verdere proces van nieuwe methoden die kunnen worden gebruikt om datalacunes op te lossen?
4. EXTRA: Wat zijn volgens u **lacunes** in de gegevens die de klinische beoordeling van ATMP's moeilijk maken?

I. Questions about the theme "HTAs"

1. What are, in your opinion, **gaps in the current HTA framework** for ATMP's used by the individual member states? (Please try to think of 3)
2. EXTRA: What are in your opinion **data gaps** which make the clinical assessment of ATMP's difficult?
3. How do developers need to cope with these uncertainties?
   1. Who do you think should all be involved in the decision to choose comparative therapy?
   2. In your opinion, how should the fact that in the pivotal studies a comparator is missing or does not correspond to a member state's practice, be dealt with?
   3. What do you think of the addition of a "**synthetic comparator**" based on a member state's historical patient data?
   4. What is your position on indirect comparisons or matching-adjusted indirect comparisons (MAIC's) as an alternative/solution to the lack of a comparator.
   5. Could you give an example of other solutions/proposals for tackling data shortages?
4. Do you think it is possible to introduce a **conditional reimbursement** after submitting new or RWE data?
   1. For example, if certain effectiveness or endpoints are reached, can a final registration automatically follow? Are there any intermediate options?
   2. The regulation states that an update/actualization is possible at the request of the members of the coordination group, can this also be done at the request of the HTD?

II. Questions about the theme "European joint clinical assessment (JCA)"

1. What is your opinion on the introduction of a European JCA, first for ATMPs and oncological medicines, then for orphan drugs and finally for all medicines?
   1. Why do you think that the European Commission choose for the **sequence of implementation** being ATMP's and oncological products (in implementation phase), orphan drugs (as of 2028) and finally all the medicinal products.
2. What will be in your opinion be the greatest **advantages of** the JCA for:
   1. HTA agency of the member states
   2. The pharmaceutical companies developing ATMPs
   3. Patients
3. What will be in your opinion be **challenges of** the JCA for:
   1. HTA agency of the member states
   2. The pharmaceutical companies developing ATMPs
   3. Patients
4. In what ways were/are you as an organization involved in developing the framework around EUnetHTA's implementation of the JCA?
5. What do you think about the introduction of the **PICO** (Patient/Intervention/Comparator/Outcomes)?
   1. In your opinion, what are the advantages of introducing PICO?
   2. In your opinion, are there any disadvantages? If so, which ones?
   3. EXTRA: Do you think that implementation of a PICO can contribute to more uniformity and **standardization** of the process?
   4. EXTRA: What other options were there next to the PICO evaluation? What made PICO stand out/be chosen above the other options on the table at that time?
6. What are the most important **changes** every **member state** needs to make in their evaluation strategies/framework before 2025 in order to be prepared to use the JCA?
7. In which areas does **EUnetHTA** need to take additional/crucial steps to ensure that implementation can be done smoothly in 2025?
   1. What problems do you expect when implementing the JCA?
   2. How could these problems be tackled?
   3. Which steps are taken to be sure that all the JCA’s will be done in time so that the patient’s access won’t be delayed or hindered?
      1. Is there enough **capacity** provided to assess all the ATMP’s in the pipeline?
   4. **How many advice** will be given in this implementation phase, from 2025 on? What is the scale up and capacity building that you pursue?
   5. **Do you have an idea of the number of ATMP’s in the pipeline? How many applications do you expect in 2025?**
   6. Why would the EUnetHTA21 consortium be the right organization to give **trainings** on the deliverables to member state and HTA bodies? Or why not?
   7. Will the assessment be published in other languages besides English or is translation something member states should provide themselves if they need it?
8. What will the impact of the JCA be on the **BENELUXA** initiative?
9. Do you think that, despite the JCA, there will continue to be **heterogeneity** between Member States in terms of impact on access for patients?
10. What do you think of the idea to combine in some way the **Scientific Advise** of EMA and the **JSC** of the coordination group? The goal is different but in essence they both look at how the clinical trials should be designed?
    1. I already had the feedback that EMA should also start conducting comparative trials and not only looking at the effect compared to a placebo. What is your opinion on this?
11. Do you think the JCA can **speed up the process of accessing ATMPs**? Why?
12. EXTRA: Do you think that in addition to the clinical assessment, a European agreement should also be sought on the degree of medical need, interpretation of a certain impact on a health outcome (e.g., very clinically relevant, clinically relevant, less clinically relevant, not relevant)?
13. EXTRA: The idea is that there will be an **earlier and more inclusive collaboration** between the different actors but how will that be implemented? At what stages/how many times/how to consider/...?
14. EXTRA: How will Europe make sure that the JCA will be **implemented/used by each member state**?
15. What are **crucial steps EUnetHTA** still needs to accomplish to have a smooth implementation?
    1. Which problems do you expect at the implementation?
    2. Will the Coordination group be ready to assess the JCA for all the ATMP's in the pipeline?
       1. Is there enough capacity provided?
    3. Which steps need to be taken to be sure that all the JCA's will be done in time so that the patient access won't be delayed or hindered?
16. EXTRA: The coordination group also considers external stakeholders/experts who can review the assessment, is that something your organization would participate on and why?

III. Questions about the theme "ATMPs"

1. What are **important challenges**, now and in the future of evaluating ATMPs?
2. Do you think the JCA will be able to **tackle these challenges**?
3. What value do you attach to **long-term RWE data** in the (re)assessment of a reimbursement?
   1. **Do you also think that further European cooperation is needed: For example, in the collection/analysis of patient data from real life?**
   2. **Would the European commission be willing to help collecting this evidence? Or the coordination of the collection?**
4. How can a reliable result regarding effectiveness and safety be decided with a **limited patient population?**
   1. How will the JCA affect this?
   2. How can companies prepare for this?
   3. Should this be discussed with the authorities?
   4. **Do you think/expect that the JCA can have an impact on the further process of new methods that can be used to solve data gaps?**

**Questions for stakeholder group 3: Pharmaceutical companies and ATMP developers**

I. Vragen over het thema "HTA's"

1. Welke **lacunes** zijn er in het huidige Belgische HTA-kader dat gehanteerd wordt in de beoordeling van een terugbetaling van ATMP’s? (probeer er minstens 3 uit te halen)
2. EXTRA: Welke **criteria** worden in België gebruikt voor de beoordeling van de klinische en economische waarde van ATMPs?
3. Hoe gaat u om met deze **onzekerheden** bij het opstellen van een dossier?
4. Hoe moeten bedrijven volgens u omgaan met deze onzekerheden?
   1. Subvraag 1: Hoe moet er volgens u omgegaan worden met het gegeven dat in de pivotale studies een comparator ontbreekt of niet overeenkomt met de Belgische praktijk?
   2. Subvraag 2: Wat vindt u van de toevoeging van een “**synthetic comparator**” op basis van Belgische historische patiënten data?
   3. Subvraag 3: Hoe staat u tegenover indirecte vergelijkingen, MAICs (matching-adjusted indirect comparisons), als alternatief/oplossing voor het gebrek van een comparator.
   4. Subvraag 3: Welke andere oplossingen/voorstellen zijn er voor het aanpakken van data tekorten?
   5. Subvraag 4: Is **vooroverleg** nodig mogelijk/nuttig om data gaps te benoemen en op voorhand afspraken te maken over hoe dit aangepakt dient te worden. Indien ja, hoe? Indien neen, waarom niet?
5. Wat zijn voor u de **data gaps** die maken dat een klinische beoordeling moeilijk maakt.
6. EXTRA: Hoe staat u tegenover de mogelijkheid om een **voorwaardelijke terugbetaling** in te voeren na het aanleveren van nieuwe of RWE data?
   1. Bijvoorbeeld als bepaalde doeltreffendheid of eindpunten worden bereikt volgt automatische een definitieve inschrijving? Zijn er tussenopties mogelijk?

II. Vragen over het thema "European joint clinical assessment " JCA

1. Wat is uw opinie over de invoering van een Europese JCA, eerst voor ATMPs en oncologische geneesmiddelen, daarna voor weesgeneesmiddelen en uiteindelijk voor alle geneesmiddelen?
2. Wat zullen volgens u de grootste **voordelen** van de JCA zijn voor:
   1. HTA organen van de lidstaten in het algemeen en België in het bijzonder?
   2. De farmaceutische bedrijven die ATMP’s ontwikkelen?
   3. Patiënten?
3. Wat zullen volgens u **uitdagingen** van de JCA zijn voor:
   1. HTA organen van de lidstaten in het algemeen en België in het bijzonder?
   2. De farmaceutische bedrijven die ATMP’s ontwikkelen?
   3. Patiënten?
4. Wat vindt u van de invoering van de **PICO** (Patient/Intervention/Comparator/Outcomes)?
   1. Wat zijn volgens u voordelen van de introductie van de PICO?
   2. Zijn er volgens u ook nadelen? Zo ja, welke?
   3. EXTRA: Denkt u dat implementatie van een PICO kan bijdragen tot meer **uniformiteit en standaardisering** van het proces?
5. Wie zou volgens u allemaal moeten betrokken worden in de keuze om de **vergelijkende therapie** te kiezen?
6. Welke **rol** kan **België** volgens u spelen tijdens het proces van de gezamenlijke klinische evaluatie?
7. Welke **stappen** moeten er volgens u nog ondernomen worden vooraleer **België** echt klaar is om de JCA te implementeren in het terugbetalingssysteem in 2025?
   1. Welke aanpassingen zijn volgens u nodig om de JCA te integreren in de Belgische terugbetalingsprocedure? Moet er iets fundamenteel veranderen aan de Belgische terugbetalingsprocedure? Zo ja, wat moet er veranderd worden?
      1. Kunt u ingaan op de uitdagingen van deze aanpassingen?
      2. Welke praktische gevolgen zullen deze veranderingen hebben voor het functioneren van de CTG?
      3. Vindt u dat er **2 verschillende comité’s** moeten opgericht worden? Bijvoorbeeld eentje die zich vooral bezig houdt met het klinische deel en bijvoorbeeld ook engageert om deel te nemen aan de JSC en JCA’s en de andere die vooral de economische evaluatie doet?
   2. Hoe zou België ervoor kunnen zorgen dat de markttoegang niet vertraagd wordt voor de patiënten? Gaat de periode van 180 dagen ingekort worden? Of gaat deze pas starten op de dag dat de JCA is gepubliceerd (max 30d na CHMP opinie)?
   3. Denkt u dat er voldoende capaciteit is/gaat zijn in België om actief deel te nemen gedurende het volledige proces van JSC tot JCA en verdere implementatie in België?
8. Welke stappen ondernemen jullie zelf in het **bedrijf/als organisatie** om klaar te zijn voor de komende wijzigingen vanaf 2025?
9. Op welke vlakken moet **EUnetHTA** nog bijkomende/cruciale **stappen** ondernemen om ervoor te zorgen dat de implementatie in 2025 vlot kan gebeuren?
   1. Subvraag 1: Welke problemen verwacht u bij het implementeren van de JCA?
   2. Subvraag 2: Hoe denkt u dat deze problemen dienen aangepast te worden?
10. Op welke manier werden/worden jullie **betrokken** bij het ontwikkelen van het kader rond de implementatie van de JCA door EUnetHTA of de coordination group?
11. Wat is het standpunt van u, ATMP ontwikkelaar over de **toegevoegde waarde/nut van JCA**?
12. Wat is de impact van JCA op het **BENELUXA** initiatief?
13. Wat vinden jullie van het idee om het scientific advise van EMA samen met de JSC te laten plaatsvinden?
14. Extra: Vindt u dat naast de klinische beoordeling er ook een Europese overeenkomst moet gezocht worden naar de **graad van medical need**, interpretatie van een bepaalde impact op een gezondheidsuitkomst (bv erg klinisch relevant, klinisch relevant, minder klinisch relevant, niet relevant)?
15. EXTRA: Het idee is dat er een eerdere en meer **inclusieve samenwerking** komt tussen de verschillende actoren, maar hoe wordt dat geïmplementeerd? In welke stadia/hoe vaak/hoe te overwegen/...?
16. EXTRA: Hoe gaat een **verschil** in implementatie van de JCA door de verschillende lidstaten (op vlak van prijsbepaling, terugbetaling etc.) **geminimaliseerd** worden?
17. EXTRA: Denkt u dat er, ondanks JCA, **heterogeniteit** zal blijven bestaan tussen lidstaten op vlak van impact op access voor patiënten?
18. EXTRA: Hoe zal de JCA gebruikt worden in het **Belgische klinische beoordelingsproces**?
    1. Subvraag 1: In welke mate ziet u de noodzaak van een herbeoordeling door de interne deskundigen van het RIZIV?
       1. Zo ja, wat zou volgens u een legitieme reden kunnen zijn voor een herevaluatie?
       2. Kunt u de meerwaarde van een eventuele herevaluatie aangeven?
       3. Kunt u ingaan op de nadelen van een herevaluatie?
    2. Subvraag 2: Is er een tussentijdse mogelijkheid om bijvoorbeeld alleen een herevaluatie te doen als de comparator in de JCA anders is dan de Belgische?

III. Vragen over het thema "ATMP's"

1. Wat zijn volgens u verder **belangrijke uitdagingen**, nu en in de toekomst van bij het evalueren van ATMP’s in België?
2. EXTRA: Denkt u dat een JCA een **oplossing** zal bieden voor deze uitdagingen?
3. Welke waarde vinden jullie dat er aan **lange termijn RWE data** moet gehecht worden in de (her)beoordeling van een terugbetaling?
   1. Op welke manier zouden jullie als bedrijf willen bijdragen aan de collectie hiervan?
4. Hoe kan er met een **beperkte patiëntenpopulatie** toch een betrouwbaar resultaat omtrent effectiviteit en veiligheid besloten worden?
   1. Subvraag 1: Op welke manier zal de JCA hierop effect hebben?
   2. **Subvraag 2:** Denkt u ook dat verdere Europese samenwerking nodig is: Bijvoorbeeld bij de collectie/analyse van patiënten data uit het echte leven?
   3. Subvraag 3: Op welke manier zouden jullie daaraan willen meewerken?
   4. Subvraag 4: Dient dit in overleg te gaan met de overheden?
   5. Subvraag 5: Denkt/verwacht u dat JCA een invloed kan hebben op het verdere proces van nieuwe methoden die kunnen gebruikt worden om data gaps op te lossen?
5. EXTRA: Denkt u dat JCA het **proces** van toegang tot ATMPs kan **versnellen**? Waarom?

**Questions for stakeholder group 4: Academics**

I. Vragen over het thema "HTA's"

1. Wat zijn volgens u lacunes in het huidige Belgische HTA-kader dat gehanteerd wordt in de beoordeling van een terugbetaling van ATMP’s? (probeer er minstens 3 uit te halen)
2. Wat is uw mening over de terugbetaling van ATMP's in het huidige systeem in België?
   1. Wat zijn volgens u optimaliseringsmaatregelen om het huidige systeem beter af te stemmen op de evaluatie van ATMP's?
3. Welke criteria worden in België gebruikt voor de beoordeling van de klinische en economische waarde van ATMPs?
4. Hoe moeten bedrijven volgens u omgaan met deze onzekerheden?
   1. Subvraag 1: Hoe moet er volgens u omgegaan worden met het gegeven dat in de pivotale studies een comparator ontbreekt of niet overeenkomt met de Belgische praktijk?
   2. Subvraag 2: Wat vindt u van de toevoeging van een “synthetic comparator” op basis van Belgische historische patiënten data?
   3. Subvraag 3: Hoe staat u tegenover indirecte vergelijkingen, MAICs (matching-adjusted indirect comparisons), als alternatief/oplossing voor het gebrek van een comparator.
   4. EXTRA: Subvraag 4: Welke andere oplossingen/voorstellen zijn er voor het aanpakken van data tekorten?
   5. EXTRA: Subvraag 5: Is vooroverleg nodig mogelijk/nuttig om data gaps te benoemen en op voorhand afspraken te maken over hoe dit aangepakt dient te worden. Indien ja, hoe? Indien neen, waarom niet?
5. Hoe staat u tegenover de mogelijkheid om een voorwaardelijke terugbetaling in te voeren na het aanleveren van nieuwe of RWE data? Bijvoorbeeld als bepaalde doeltreffendheid of eindpunten worden bereikt volgt een automatische een definitieve inschrijving? Zijn er tussenopties mogelijk?

II. Vragen over het thema "European joint clinical assessment "JCA""

1. Wat is uw opinie over de invoering van een Europese JCA, eerst voor ATMPs en oncologische geneesmiddelen, daarna voor weesgeneesmiddelen en uiteindelijk voor alle geneesmiddelen?
2. Wat zullen volgens u de grootste voordelen van de JCA zijn voor:
   1. HTA organen van de lidstaten in het algemeen en België in het bijzonder?
   2. De farmaceutische bedrijven die ATMP’s ontwikkelen?
   3. Patiënten?
3. Wat zullen volgens u uitdagingen van de JCA zijn voor:
   1. HTA organen van de lidstaten in het algemeen en België in het bijzonder?
   2. De farmaceutische bedrijven die ATMP’s ontwikkelen?
   3. Patiënten?
4. Wat vindt u van de invoering van de PICO (Patient/Intervention/Comparator/Outcomes)?
   1. Welke nadelen zou u toewijzen aan het gebruik van de PICO?
   2. Op welke manier denkt u dat de invoering van de PICO kan bijdragen tot meer uniformiteit en standaardisering van het proces?
5. Welke rol kan België volgens u spelen tijdens het proces van de gezamenlijke klinische evaluatie?
6. Hoe zal de JCA gebruikt worden in het Belgische klinische beoordelingsproces?
   1. Gaat er een herevaluatie gebeuren door de interne experten van het RIZIV?
      1. Indien wel, wat is de reden van een herevaluatie?
   2. Welke aanpassingen zijn volgens u nodig om de JCA te integreren in de Belgische terugbetalingsprocedure? Moet er iets fundamenteel veranderen aan de Belgische terugbetalingsprocedure? Zo ja, wat moet er veranderd worden?
      1. Kunt u ingaan op de uitdagingen van deze aanpassingen?
      2. Welke praktische gevolgen zullen deze veranderingen hebben voor het functioneren van de CTG?
      3. Vindt u dat er 2 verschillende comité’s moeten opgericht worden? Bijvoorbeeld eentje die zich vooral bezig houdt met het klinische deel en bijvoorbeeld ook engageert om deel te nemen aan de JSC en JCA’s en de andere die vooral de economische evaluatie/BI analyseert?
   3. Hoe zou België ervoor kunnen zorgen dat de markttoegang niet vertraagd wordt voor de patiënten? Gaat de periode van 180 dagen ingekort worden? Of gaat deze pas starten op de dag dat de JCA is gepubliceerd (max 30d na CHMP opinie)?
   4. EXTRA: Is er een tussenmogelijkheid om bijvoorbeeld enkel een herevaluatie te doen indien de comparator gebruikt in het JCA is verschillend aan de Belgische?
7. Welke stappen moeten er volgens u nog ondernomen worden vooraleer België echt klaar is om de JCA te implementeren in het terugbetalingssysteem in 2025?
   1. Welke rol zou u hierin willen opnemen?
8. Op welke vlakken moet EUnetHTA nog bijkomende/cruciale stappen ondernemen om ervoor te zorgen dat de implementatie in 2025 vlot kan gebeuren?
   1. EXTRA: Welke problemen verwacht u bij het implementeren van de JCA?
   2. EXTRA: Hoe denkt u dat deze problemen dienen aangepast te worden?
9. Wat is het standpunt van u, uw universitaire omgeving over de toegevoegde waarde/nut van JCA?
10. Wat is de impact van JCA op het BENELUXA initiatief en andere cross-country collaborations?
11. Wat vindt u van het feit dat de assessors voor de JSC en de assessors voor de JCA verschillend moeten zijn?
12. EXTRA: Vindt u dat naast de klinische beoordeling er ook een Europese overeenkomst moet gezocht worden naar de graad van medical need, interpretatie van een bepaalde impact op een gezondheidsuitkomst (bv erg klinisch relevant, klinisch relevant, minder klinisch relevant, niet relevant)?
13. EXTRA: Hoe gaat een verschil in implementatie van de JCA door de verschillende lidstaten (op vlak van prijsbepaling, terugbetaling etc.) geminimaliseerd worden?
14. EXTRA: Denkt u dat er, ondanks JCA, heterogeniteit zal blijven bestaan tussen lidstaten op vlak van impact op access voor patiënten?
15. EXTRA: Wie zou volgens u allemaal moeten betrokken worden in de keuze om de vergelijkende therapie te kiezen?

III. Vragen over het thema "ATMP's"

1. Wat zijn volgens u verder belangrijke uitdagingen, nu en in de toekomst van bij het evalueren van ATMP’s in België?
2. EXTRA: Denkt u dat een JCA een oplossing zal bieden voor deze uitdagingen?
3. Denkt u dat JCA het proces van toegang tot ATMPs kan versnellen? Waarom?
4. Hoe kan er met een beperkte patiëntenpopulatie toch een betrouwbaar resultaat omtrent effectiviteit en veiligheid besloten worden?
   1. Subvraag 1: Op welke manier zal de JCA hierop effect hebben?
   2. Denkt u ook dat verdere Europese samenwerking nodig is: Bijvoorbeeld bij de collectie/analyse van patiënten data uit het echte leven?
      1. Wie moet volgens u dan allemaal deel uit maken van deze samenwerking?
   3. Subvraag 3: Hoe kunnen bedrijven zicht hierop voorbereiden?
   4. Subvraag 4: Dient dit in overleg te gaan met de overheden?
   5. Subvraag 5: Denkt/verwacht u dat JCA een invloed kunnen hebben op het verdere proces van nieuwe methoden die kunnen gebruikt worden data gaps op te lossen?
5. Welke waarde hecht u aan lange termijn RWE data in de (her)beoordeling van een terugbetaling?
6. EXTRA: Wat is volgens u de reden waarom dat de JCA eerst wordt gebruikt voor ATMP’s en oncologische producten en pas later voor andere geneesmiddelen?

**Questions for stakeholder group 5: Health insurance and sick funds**

I. Vragen over het thema "HTA's"

1. Wat zijn volgens u lacunes in het huidige Belgische HTA-kader dat gehanteerd wordt in de beoordeling van een terugbetaling van ATMP’s? (probeer er minstens 3 uit te halen)
2. Wat is uw mening over de terugbetaling van ATMP's in het huidige systeem in België?
   1. Wat zijn volgens u optimaliseringsmaatregelen om het huidige systeem beter af te stemmen op de evaluatie van ATMP's?
3. Wat zijn voor u de data gaps die maken dat een klinische beoordeling moeilijk maakt.
   1. EXTRA: Hoe gaat u zelf om met deze onzekerheden in uw beoordeling?
4. Hoe moeten bedrijven volgens u omgaan met deze onzekerheden?
   1. Subvraag 1: Hoe moet er volgens u omgegaan worden met het gegeven dat in de pivotale studies een comparator ontbreekt of niet overeenkomt met de Belgische praktijk?
   2. Subvraag 2: Wat vindt u van de toevoeging van een “synthetic comparator” op basis van Belgische historische patiënten data?
   3. Subvraag 3: Hoe staat u tegenover indirecte vergelijkingen, MAICs (matching-adjusted indirect comparisons), als alternatief/oplossing voor het gebrek van een comparator.
   4. Subvraag 4: Welke andere oplossingen/voorstellen zijn er voor het aanpakken van data tekorten?
   5. EXTRA: Subvraag 5: Is vooroverleg nodig mogelijk/nuttig om data gaps te benoemen en op voorhand afspraken te maken over hoe dit aangepakt dient te worden. Indien ja, hoe? Indien neen, waarom niet?
5. EXTRA: Hoe staat u tegenover de mogelijkheid om een voorwaardelijke terugbetaling in te voeren na het aanleveren van nieuwe of RWE data? Bijvoorbeeld als bepaalde doeltreffendheid of eindpunten worden bereikt volgt een automatische een definitieve inschrijving? Zijn er tussenopties mogelijk?

II. Vragen over het thema "European joint clinical assessment "JCA""

1. Wat is uw opinie over de invoering van een Europese JCA, eerst voor ATMPs en oncologische geneesmiddelen, daarna voor weesgeneesmiddelen en uiteindelijk voor alle geneesmiddelen?
2. Wat zullen volgens u de grootste voordelen van de JCA zijn voor:
   1. HTA organen van de lidstaten in het algemeen en België in het bijzonder?
   2. De farmaceutische bedrijven die ATMP’s ontwikkelen?
   3. Patiënten?
3. Wat zullen volgens u uitdagingen van de JCA zijn voor:
   1. HTA organen van de lidstaten in het algemeen en België in het bijzonder?
   2. De farmaceutische bedrijven die ATMP’s ontwikkelen?
   3. Patiënten?
4. Wat vindt u van de invoering van de PICO (Patient/Intervention/Comparator/Outcomes)?
5. EXTRA: Denkt u dat implementatie van een PICO kan bijdragen tot meer uniformisering en standaardisering van het proces?
6. Wat is het standpunt van u, uw organisatie over de toegevoegde waarde/nut van JCA?
7. Welke rol kan België volgens u spelen tijdens het proces van de gezamenlijke klinische evaluatie?
8. Hoe zal de JCA gebruikt worden in het Belgische klinische beoordelingsproces?
   1. Subvraag 1: In welke mate ziet u de noodzaak van een herbeoordeling door de interne deskundigen van het RIZIV?
      1. Zo ja, wat zou volgens u een legitieme reden kunnen zijn voor een herevaluatie?
      2. Kunt u de meerwaarde van een eventuele herevaluatie aangeven?
      3. Kunt u ingaan op de nadelen van een herevaluatie?
   2. Subvraag 2: Is er een tussentijdse mogelijkheid om bijvoorbeeld alleen een herevaluatie te doen als de comparator in de JCA anders is dan de Belgische?
   3. Subvraag 3: Welke aanpassingen zijn volgens u nodig om de JCA te integreren in de Belgische terugbetalingsprocedure? Moet er iets fundamenteel veranderen aan de Belgische terugbetalingsprocedure? Zo ja, wat moet er veranderd worden?
      1. Kunt u ingaan op de uitdagingen van deze aanpassingen?
      2. Welke praktische gevolgen zullen deze veranderingen hebben voor het functioneren van de CTG?
      3. Vindt u dat er 2 verschillende comité’s moeten opgericht worden? Bijvoorbeeld eentje die zich vooral bezig houdt met het klinische deel en bijvoorbeeld ook engageert om deel te nemen aan de JSC en JCA’s en de andere die vooral de economische evaluatie/BI analyseert?
   4. Hoe zou België ervoor kunnen zorgen dat de markttoegang niet vertraagd wordt voor de patiënten? Gaat de periode van 180 dagen ingekort worden? Of gaat deze pas starten op de dag dat de JCA is gepubliceerd (max 30d na CHMP opinie)?
9. Welke stappen moeten er volgens u nog ondernomen worden vooraleer België echt klaar is om de JCA te implementeren in het terugbetalingssysteem in 2025?
   1. Welke rol zou u hierin willen opnemen?
10. Op welke vlakken moet EUnetHTA nog bijkomende/cruciale stappen ondernemen om ervoor te zorgen dat de implementatie in 2025 vlot kan gebeuren?
    1. Subvraag 1: Welke problemen verwacht u bij de implementatie?
    2. Subvraag 2: Hoe kunnen deze problemen worden aangepakt?
    3. Subvraag 3: Zal EUnetHTA klaar zijn om de JCA te beoordelen voor alle ATMP's in de pijplijn?
    4. Subvraag 4: Weet u hoeveel adviezen er zullen worden gegeven in 2025?
       1. Wat is de opschaling en capaciteitsopbouw die u doorzet?
    5. Subvraag 5: Welke stappen worden er genomen om er zeker van te zijn dat alle JCA's op tijd worden gedaan, zodat de toegang van de patiënt niet wordt vertraagd of belemmerd?
    6. Subvraag 6: Zullen er vertalingen voorzien worden van de assessment?
    7. Wat vindt u van het feit dat de assessors van de JSC verschillend moeten zijn van de assessors van de JCA?
11. Wat is de impact van JCA op het BENELUXA initiatief?
12. EXTRA: Vindt u dat naast de klinische beoordeling er ook een Europese overeenkomst moet gezocht worden naar de graad van medical need, interpretatie van een bepaalde impact op een gezondheidsuitkomst (bv erg klinisch relevant, klinisch relevant, minder klinisch relevant, niet relevant)?
13. EXTRA: Hoe gaat een verschil in implementatie van de JCA door de verschillende lidstaten (op vlak van prijsbepaling, terugbetaling etc.) geminimaliseerd worden?
14. EXTRA: Denkt u dat er, ondanks JCA, heterogeniteit zal blijven bestaan tussen lidstaten op vlak van impact op access voor patiënten?
15. EXTRA: Wie zou volgens u allemaal moeten betrokken worden in de keuze om de vergelijkende therapie te kiezen?

III. Vragen over het thema "ATMP's"

1. Wat zijn volgens u verder belangrijke uitdagingen, nu en in de toekomst van bij het evalueren van ATMP’s in België?
2. EXTRA: Denkt u dat een JCA een oplossing zal bieden voor deze uitdagingen?
3. Hoe kan er met een beperkte patiëntenpopulatie toch een betrouwbaar resultaat omtrent effectiviteit en veiligheid besloten worden?
   1. Subvraag 1: Op welke manier zal de JCA hierop effect hebben?
   2. Subvraag 2: Denkt u ook dat verdere Europese samenwerking nodig is: Bijvoorbeeld bij de collectie/analyse van patiënten data uit het echte leven?
   3. Subvraag 3: Hoe kunnen bedrijven zicht hierop voorbereiden?
   4. Subvraag 4: Dient dit in overleg te gaan met de overheden?
   5. Subvraag 5: Denkt/verwacht u dat JCA een invloed kunnen hebben op het verdere proces van nieuwe methoden die kunnen gebruikt worden data gaps op te lossen?
4. Welke waarde hecht u aan lange termijn RWE data in de (her)beoordeling van een terugbetaling?
5. EXTRA: Denkt u dat JCA het proces van toegang tot ATMPs kan versnellen? Waarom?
6. EXTRA: Wat is volgens u de reden waarom dat de JCA eerst wordt gebruikt voor ATMP’s en oncologische producten en pas later voor andere geneesmiddelen?

# Supplementary Figures and Tables

## Supplementary Figures

##
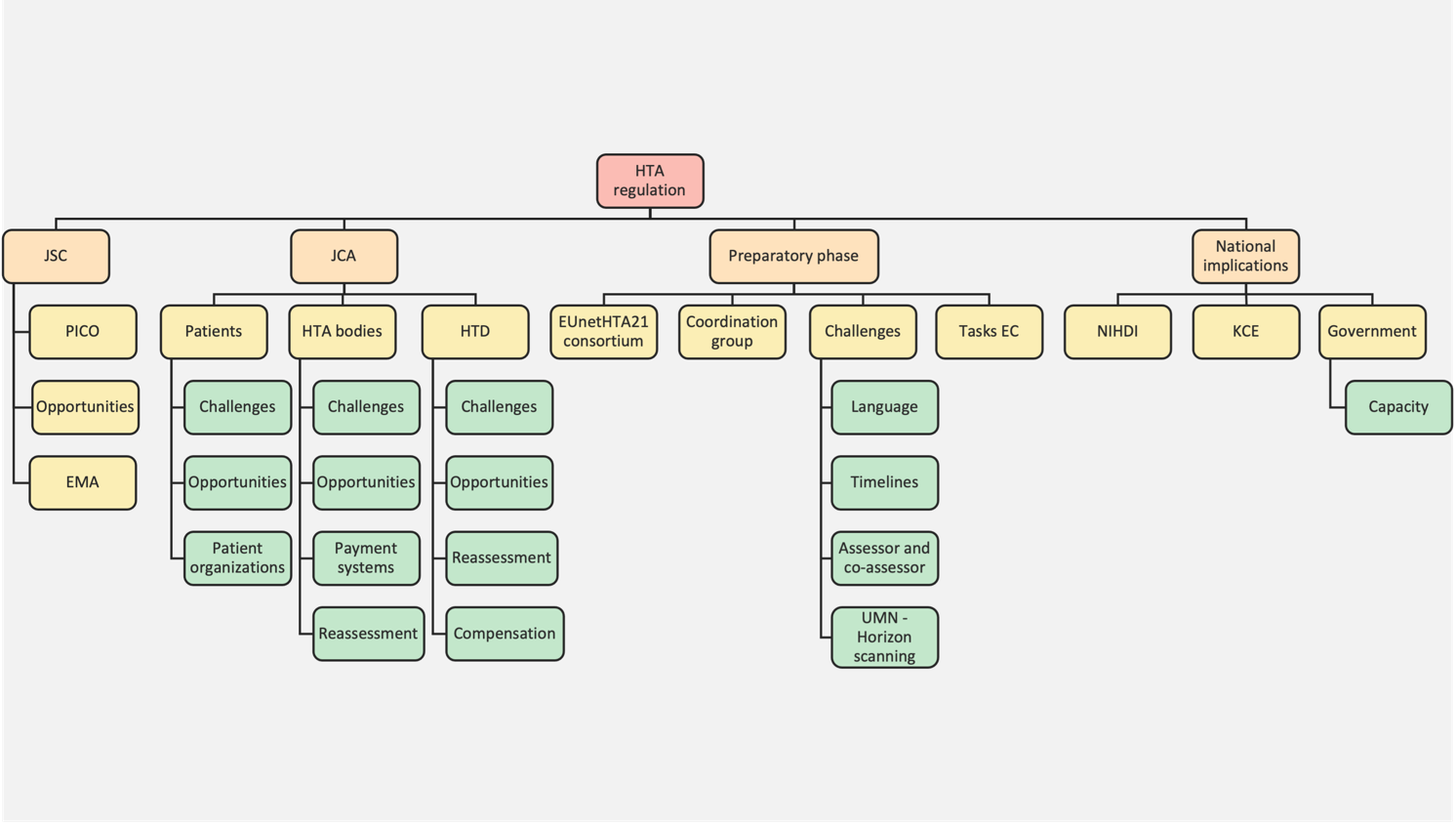
Supplementary Figure 1. Decision tree one.


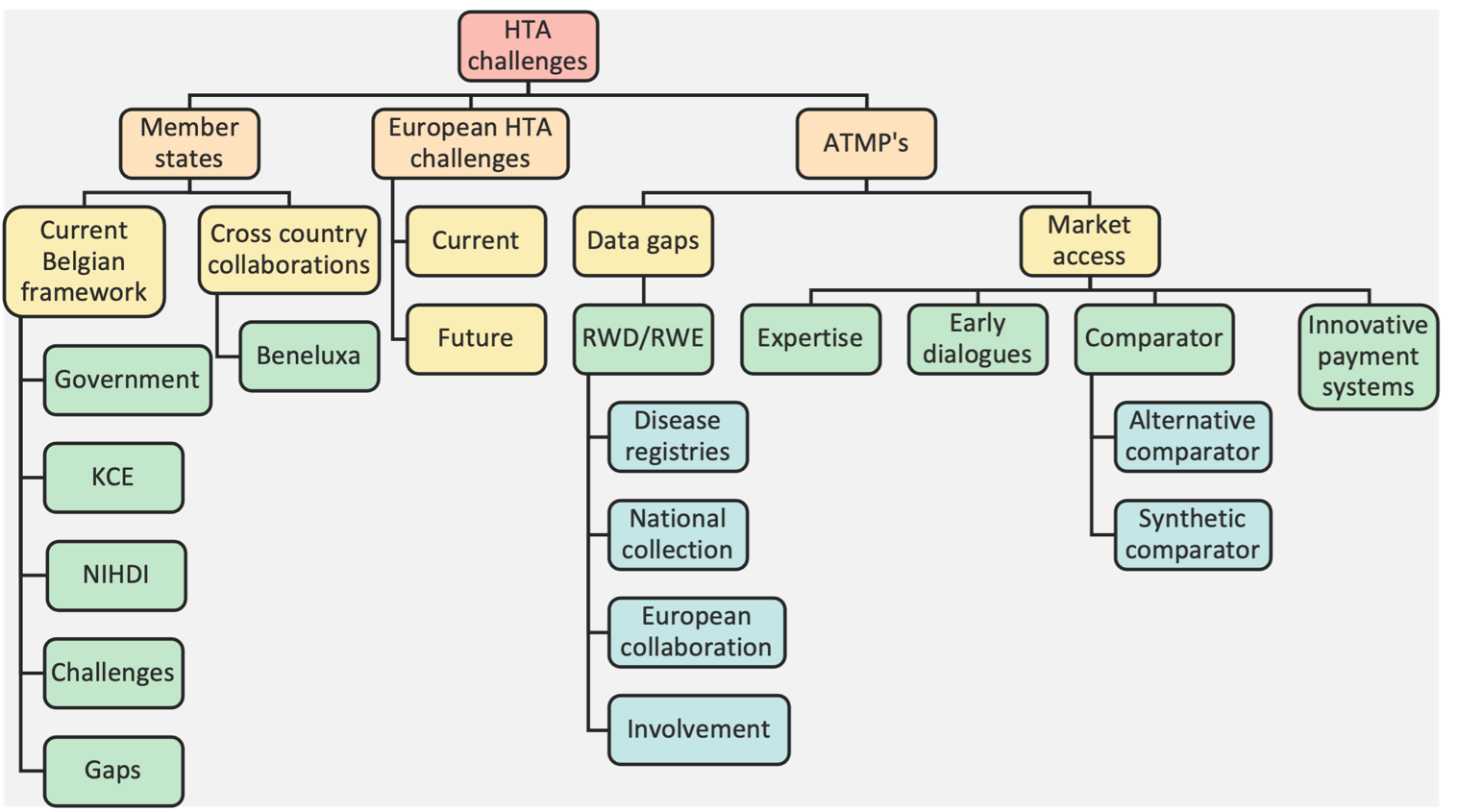


**Supplementary Figure 2.** Decision tree two.
